# HIV Protective KIR3DL1/S1-HLA-B Genotypes Influence NK Cell-Mediated Inhibition of HIV Replication in Autologous CD4 Targets

**DOI:** 10.1371/journal.ppat.1003867

**Published:** 2014-01-16

**Authors:** Rujun Song, Irene Lisovsky, Bertrand Lebouché, Jean-Pierre Routy, Julie Bruneau, Nicole F. Bernard

**Affiliations:** 1 Research Institute of the McGill University Health Centre, Montreal, Quebec, Canada; 2 Division of Experimental Medicine, McGill University, Montreal, Quebec, Canada; 3 Chronic Viral Illness Service, McGill University Health Centre, Montreal, Quebec, Canada; 4 Department of Family Medicine, McGill University, Montreal, Quebec, Canada; 5 Division of Hematology, Royal Victoria Hospital, McGill University Health Centre, Montreal, Quebec, Canada; 6 Centre de Recherche du Centre Hospitalier de l'Université de Montréal, Montreal, Quebec, Canada; 7 Department of Family Medicine, Université de Montréal, Montreal, Quebec, Canada; 8 Division of Clinical Immunology, McGill University Health Centre, Montreal, Quebec, Canada; Miller School of Medicine, United States of America

## Abstract

Carriage of the genetic combination encoding a high expression inhibitory Killer Immunoglobulin-like Receptor (KIR)3DL1 with its ligand, HLA-B*57 (*h/*y+B*57) is associated with slower time to AIDS and better HIV viral load control than being a *Bw6* homozygote (*Bw6hmz*). Natural Killer (NK) cells from *h/*y+B*57 carriers receive potent educational signals through HLA-B*57 KIR3DL1 ligation leading to high functional potential. NK cells from *Bw6hmz* are not educated through KIR3DL1 because Bw6 antigens do not interact with this inhibitory receptor. To better understand the impact of KIR/HLA combinations on NK cell mediated anti-viral activity we measured NK cell mediated inhibition of HIV replication in autologous infected CD4 (iCD4) cells by assessing the frequency of p24 positive CD4 targets and supernatant levels of HIV p24 longitudinally in the presence versus absence of NK cells. Forty-seven HIV uninfected subjects were studied, including carriers of *h/*y+B*57, a low expression *KIR3DL1* genotype with HLA-B*57 termed *l/*x+B*57, a genotype designated 3DS1+*80I and *Bw6hmz*. NK cells from *h/*y+B*57 carriers, like those from 3DS1+*80I subjects, inhibited HIV replication in autologous iCD4 cells better than those from *Bw6hmz* and *l/*x+B*57 carriers. Cell contact between NK and iCD4 cells activated NK cells to inhibit viral replication in a non-contact dependent fashion through secretion of CC-chemokines. iCD4 stimulated NK cells from *h/*y+B*57 and 3DS1+*80I carriers produced higher levels of CC-chemokines than those from *Bw6hmz* or *l/*x+B*57 carriers. Higher levels of CC-chemokines were produced by KIR3DL1^+^ than KIR3DL1^−^ NK cells. We conclude that NK-mediated inhibition of viral replication in autologous iCD4 cells is partially due to a block at the level of HIV entry into new targets by secreted CC-chemokines.

## Author Summary

Natural Killer (NK) cells function in anti-tumor and anti-viral defenses, including those directed against HIV. HIV infected cells can activate NK cells, which, once activated, inhibit HIV replication in infected targets. NK cell activation levels depend on the interaction of cell surface receptors on NK cells with the molecules (or ligands) they recognize on neighboring target cells. One receptor-ligand combination has been identified to have a strong effect on slowing time to AIDS, HIV viral load control and NK cell activation potential. We compared anti-HIV NK cell responses in individuals with this NK receptor-ligand combination to those from subjects having NK receptor-ligand combinations associated a neutral effect on time to AIDS. NK cells inhibited HIV replication in autologous infected cells more potently when they came from individuals with NK receptor-ligand (KIR/HLA) gene combinations associated with slow versus typical time to AIDS. Inhibition of HIV replication was due to secretion of factors (chemokines) that bind and block the co-receptor HIV uses to enter susceptible target cells. NK cells from subjects with KIR/HLA combinations associated with potent NK cell anti-HIV activity secreted more chemokines than those from subjects with KIR/HLA combinations associated with weak anti-HIV NK cell activity.

## Introduction

NK cells function in innate immune responses to transformed and virally infected cells. They can exert their anti-viral effects soon after encountering infected targets without prior sensitization [1]. NK cell function is determined by signals from activating and inhibitory cell surface receptors, which include Killer Immunoglobulin-like Receptors (KIR) [2]. Among these are inhibitory KIR3DL1 (3DL1) and activating KIR3DS1 (3DS1) receptors, which are encoded by alleles at the same *KIR3DL1/S1* locus [3]. 3DL1 receptors can be classified into those expressed on NK cell surfaces at high levels (*h) low levels (*l) or *004, which is only transiently expressed [4]–[7]. 3DL1 homozygous genotypes can be dichotomized into *h/*y and *l/*x groups where *h/*y genotypes encode receptors expressed on the NK cell surface at higher levels than those encoded by *l/*x genotypes [6].

Epidemiological studies have found that several 3DL1 homozygous genotypes co-carried with a subset of *HLA-B* and *–A* alleles belonging to the *HLA-Bw4* group are associated with slower time to AIDS and viral load (VL) control [7]. HLA-Bw4 antigens differ from the remaining HLA-Bw6 (Bw6) antigens by amino acids at positions 77–83 [8]. The genotype combinations that confers the highest degree of protection in terms of time to AIDS and VL control is 3DL1*h/*y co-carried with HLA-B*57 (*h/*y+B*57) [7]. Subjects with this combined genotype are more frequent among HIV Exposed Seronegative (HESN) than HIV susceptible individuals, implicating carriage of this genotype combination in reducing HIV infection risk [9]. NK cells from carriers of *h/*y+B*57 have more potent NK cell functional potential as defined by HLA-null cell induced secretion of IFN-γ and TNF-α and expression of CD107a, a marker for degranulation, than those from carriers of the receptor or ligand alone, including those from carriers of the *l/*x+B*57 KIR/HLA genotype and *Bw6* homozygotes (*Bw6hmz*) [9], [10]. Bw6 antigens do not interact with 3DL1 receptors and are thus unable to educate NK cells through this inhibitory NK receptor [11], [12]. NK cell education is an ontological process that depends on the interaction of inhibitory NK receptors, such as 3DL1, with their MHC class I (MHC-1) ligands. The strength of educational signals received during NK cell development determines NK cell functional potential [11], [13], [14]. Thus, NK cells from *l/*x+B*57 carriers may be less functional than those from *h/*y+B*57 positive subjects since the former express less 3DL1 than the later and thus receive lower level educational signals upon interaction with the same ligand [6], [10], [15].

The KIR/HLA combination 3DS1 co-expressed with a Bw4 antigen having an isoleucine at position 80 of the HLA heavy chain (3DS1+*80I) is also associated with slower time to AIDS and VL control [16], [17]. NK cells from carriers of the 3DS1+*80I genotype inhibit viral replication in autologous HIV-infected CD4 (iCD4) T cells more potently than those from individuals carrying the receptor or ligand alone, or neither [18]. Together, these functional studies suggest that the association of certain *KIR/HLA* genotypes with either protection from HIV infection in HESN subjects or slow time to AIDS and VL control in those who are HIV infected, is linked to NK cell function.

How NK cells inhibit viral replication in autologous CD4 T cells is not completely understood.

One possibility is through the secretion of the CC-chemokines CCL3, CCL4, and CCL5 upon activation following recognition of autologous HIV iCD4 cells. These chemokines can suppress HIV replication by competing with the virus for binding the CCR5 co-receptor and blocking HIV entry into CD4 cells [19], [20]. In this report we investigated whether NK cells from individuals carrying *h/*y+B*57 inhibited HIV replication in autologous HIV iCD4 cells better than those from *l/*x+B*57 carriers and *Bw6hmz*. The cell contact requirement for inhibition of viral replication was assessed. We also measured the production of CC-chemokines by NK cells stimulated with autologous HIV iCD4 cells. We determined whether CC-chemokine secretion levels differed based on the *KIR/HLA* genotype and evaluated the effect of CC-chemokine neutralization on NK cell mediated inhibition of HIV replication.

## Results

### NK cells inhibit HIV viral replication in autologous iCD4 T cells

CD4 cells from 17 individuals were infected with HIV and co-cultured with or without autologous NK cells at an NK∶iCD4 ratio of 10∶1 and 1∶1. Fig. S1 shows that NK cells inhibited viral replication, at all times tested and at both NK∶iCD4 ratios. For the 10∶1 and 1∶1 NK∶iCD4 cell ratios there were no significant between time point differences in viral inhibition (p = 0.15 and p = 0.42, Friedman test). Viral inhibition was significantly higher at days 7 and 10 for the 10∶1 versus 1∶1 NK∶iCD4 ratio (p = 0.51, 0.002 and 0.008 for days 3, 7 and 10, respectively, Mann-Whitney test). The higher viral inhibition levels seen in wells containing NK and iCD4 cells at a 10∶1 ratio compared to a 1∶1 ratio or for iCD4 cells cultured alone could have been due to differences in the number of cells in these culture conditions. For example, it was possible that higher cell numbers limited cell survival and this is what led to inhibition of HIV replication. To rule out this possibility we compared the number of live CD4 cells present at days 7 and 10 of culture between conditions where NK and iCD4 were cultured at 10∶1 with those where iCD4 were cultured alone. No significant differences in CD4 numbers were found (not shown). Based on the 10∶1 NK∶iCD4 T cell ratio showing more potent inhibition of viral replication than the 1∶1 ratio, we used the 10∶1 ratio for subsequent experiments.

### NK-iCD4 cell contact contributes to NK cell mediated inhibition of HIV replication

**Figure 1:**
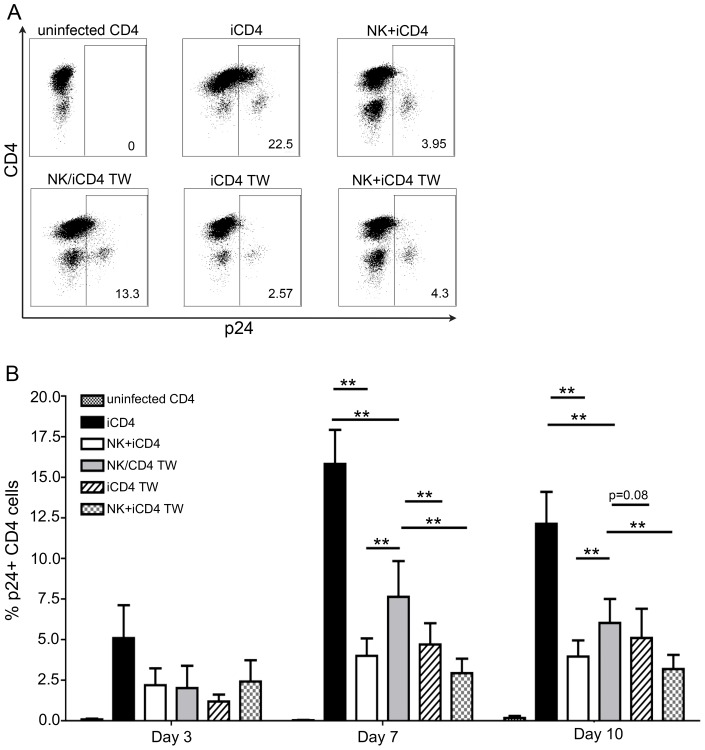
NK cells inhibit HIV replication in autologous HIV infected CD4 T cells in a contact dependent manner. (A) Flow plots show the frequency of p24 positive CD4 cells from a single individual cultured for 7 days under the following conditions: uninfected CD4 T cells cultured alone, infected CD4 (iCD4) cells cultured alone, iCD4 cells cultured with autologous NK cells in the same well at a 10∶1 NK∶iCD4 ratio (NK+iCD4), iCD4 cells and NK cells cultured in separate transwell chambers at a 10∶1 NK∶iCD4 ratio (NK/iCD4 TW), iCD4 cells cultured alone in the upper chamber of a transwell with NK cells and iCD4 cells cultured together in the lower transwell chamber at a 10∶1 NK∶iCD4 ratio (iCD4 TW), iCD4 cells cultured with NK cells in the same transwell chamber at a 10∶1 NK∶iCD4 cell ratio (NK+iCD4 TW). (B) Bar graphs show the frequency of HIV infected cells on days 3, 7 and 10 under the same culture conditions as described in (A) for up to 12 individuals. One subject was positive for *h/*y+B*57, 7 were 3DS1+*80I, 2 were *Bw6hmz*, 1 was 3DS1+Bw4 not *80I and 1 was 3DL1hmz+*80I (not B*57). Bar height and error bars represent the mean and the standard error of the mean for each group. Lines linking bars indicate comparisons where means are significantly different. “*” = a p-value<0.05, “**” = a p-value of <0.01.

NK cell mediated inhibition of viral replication was assessed by measuring the frequency of intracellular HIV-Gag-p24 positive CD4 cells using anti-p24 specific KC57 monoclonal antibody (mAb). Fig. S2 shows the gating strategy used to assess the percent of p24 positive CD4 cells. Fig. 1A depicts flow cytometry plots showing the frequency of p24 positive CD4 cells at day 7 for several culture conditions for a single individual. Fig. 1B shows longitudinal results for up to 12 subjects, 5 on day 3, 10 on day 7 and 12 on day 10. In the presence of NK cells (NK+iCD4) the frequency of p24 positive CD4 cells was lower than that in cultures of iCD4 cells alone (p = 0.18, p = 0.002 and p<0.001 for day 3, 7 and 10, respectively, Wilcoxon matched-pairs test). When NK and iCD4 cells were cultured in different transwell chambers (NK/iCD4 TW), which prevents NK and iCD4 cell contact, the frequency of p24 positive CD4 cells was significantly higher than in conditions where NK and iCD4 were cultured together either in regular wells or the same chamber of a transwell (NK+iCD4 or NK+iCD4 TW versus NK/iCD4 TW, p<0.05 for all comparisons at days 7 and 10, Wilcoxon). However, the frequency of p24+ CD4 cells in the NK/iCD4 TW condition remained below that observed in iCD4 cells (p≤0.002 for comparisons at days 7 and 10). These results implicate NK iCD4 cell contact as a contributing factor in suppression of virus spread. However, since abrogating NK and iCD4 contact does not return the percent of p24 positive CD4 cells to levels seen in iCD4 cells cultured alone, non-contact dependent mechanisms are also likely involved in NK cell mediated inhibition of HIV replication.

If iCD4 cells and co-cultures of NK and iCD4 cells are incubated in upper and lower transwell chambers, respectively, the frequency of p24 positive CD4 cells in the upper chamber (iCD4 TW) is lower than that seen when only NK cells are present in the lower chamber (NK/iCD4 TW) (p = 0.007 and p = 0.08 for days 7 and 10, Wilcoxon). These results suggest that contact between NK and autologous HIV iCD4 cells produces soluble factors that can then suppress HIV spread in the same well or cross a transwell membrane to suppress the spread of HIV in iCD4 cells physically separated from NK cells.

### NK cells produce CC-chemokines in response to stimulation with autologous HIV iCD4 cells

We questioned whether autologous iCD4 cells could activate NK cells to secrete CC-chemokines. We reasoned that if this were the case, these soluble factors could be responsible for inhibiting HIV replication under conditions where iCD4 are either co-cultured with NK cells or in a separate transwell chamber from NK+iCD4 cultures.

**Figure 2:**
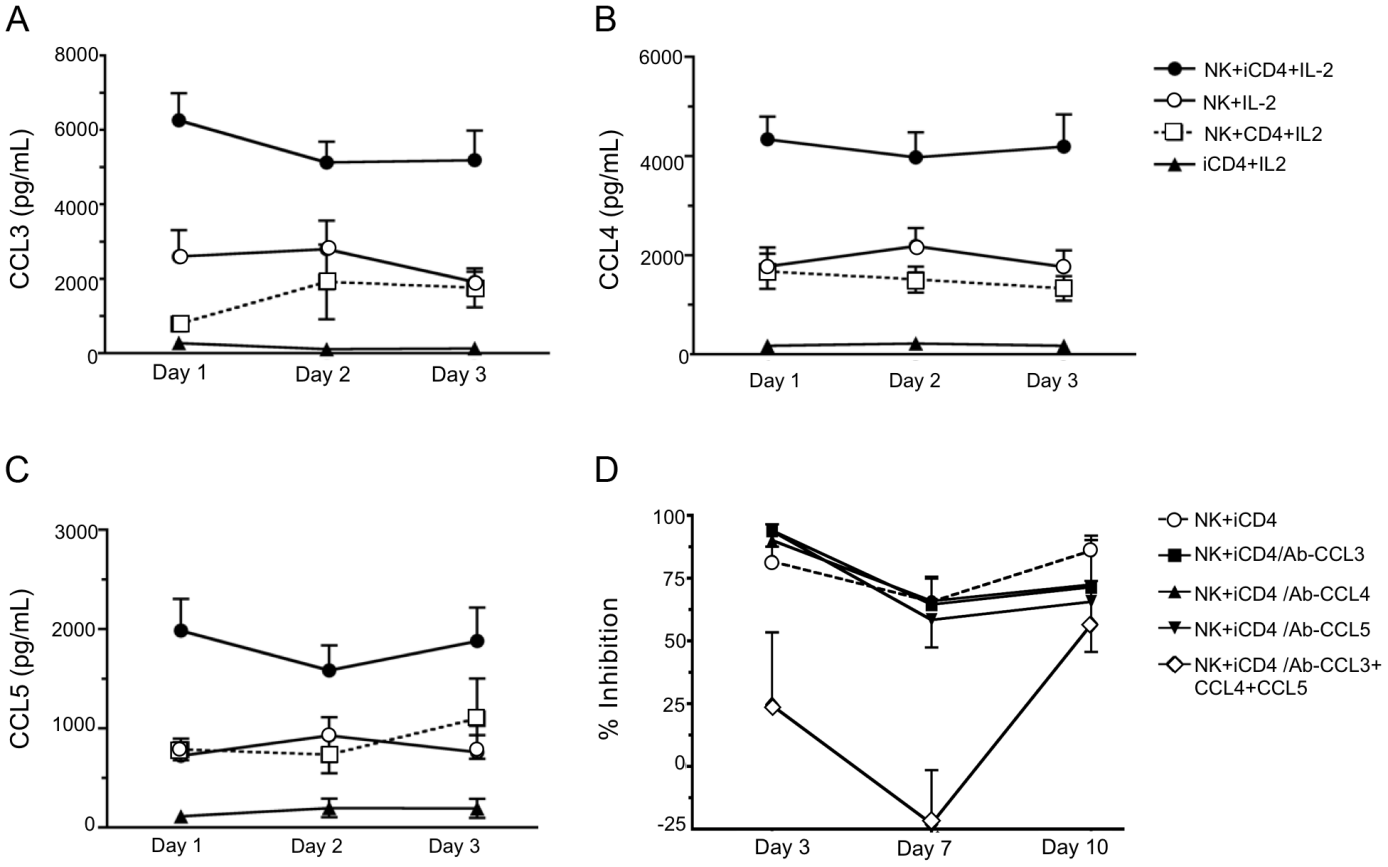
Infected CD4 T cells stimulate autologous NK cell to produce CC-chemokines. Levels of CCL3 (A), CCL4 (B) and CCL5 (C) secreted into supernatants after days 1, 2 and 3 under various culture conditions were assessed by ELISA. Shown are results for NK cells cultured with infected CD4 cells (iCD4) at a 10∶1 ratio with 100 international units (IU)/ml of human recombinant IL-2 (IL-2) (NK+iCD4+IL-2, n = 33 observations), NK cells cultured with 100 IU/ml IL-2 (NK+IL-2, n = 24 observations), NK cells cultured with uninfected CD4 and 100 IU/ml of IL-2 (NK+CD4+IL-2, n =  9 observations) and iCD4 with 100 IU/ml IL-2 (iCD4+IL-2, n = 11 observations). Data points and error bars represent the mean and the standard error of the mean for the groups. Results were generated using subjects with the following KIR/HLA genotypes; *h/*y+B*57 (n = 4), 3DS1+*80I (n = 5), *Bw6hmz* (n = 4), *l/*x+B*57 (n = 4) and other *KIR/HLA* (n = 5). Samples were tested on up to 4 occasions in separate experiments. (D) Percent inhibition of viral replication on days 3, 7 and 10 of an NK cell autologous iCD4 cell co-culture (10∶1 ratio) in the absence of anti-CC-chemokine neutralizing antibodies (nAbs), in the presence of anti-CCL3, anti-CCL4 or anti-CCL5 nAbs, individually, or together. Results were generated using subjects with the following *KIR/HLA* gentotypes; *h/*y+B*57 (n = 2) and 3DS1+*80I (n = 5). Data points and error bars represent the mean and standard error of the mean of values for the groups.

We assessed CC-chemokine secretion under several conditions at days 1, 2 and 3 of culture. Fig. 2A–C show that PHA stimulated HIV iCD4 cells co-cultured with NK cells and recombinant human IL-2 (IL-2) (NK+iCD4+IL-2) produced CCL3, CCL4 and CCL5 at higher levels than do either NK cells alone with IL-2 (NK+IL-2), NK cells cultured with uninfected CD4 cells and IL-2 (NK+CD4+IL-2) or iCD4 cells with IL-2 (iCD4+IL-2) (p<0.05 for all CC-chemokines on each day tested, Kruskal-Wallis test). All pair-wise comparisons between CC-chemokine levels secreted in the NK+iCD4+IL-2 condition and those in each of the other 3 conditions were statistically significant, except for those between NK+iCD4+IL-2 and NK+CD4+IL-2 for CCL5 at days 1, 2 and 3 (p = 0.07, 0.25 and 0.34, respectively, Dunn's post-test comparisons). NK cells cultured without IL-2 and CD4 cells, whether PHA stimulated or not, HIV infected or not and cultured with or without IL-2 produced low levels of the 3 CC-chemokines in the range of 200 pg/ml or lower (data not shown). Thus, NK cells stimulated by autologous HIV iCD4 cells and IL-2 are a source of secreted CC-chemokines and produce more CC-chemokines than NK cells or iCD4 cells alone culture media containing IL-2.

### Inhibition of HIV replication by NK cells can be reversed by neutralizing anti-CCL3, CCL4 and CCL5 antibodies (Abs)

To confirm that CC-chemokines contribute to inhibition of HIV replication, neutralizing Abs to each CC-chemokine were added to iCD4 cells at the same time as NK cells. As seen in Fig. 2D for percent inhibition of viral replication compared to iCD4 cells alone, the addition of neutralizing Abs to individual CC-chemokines had no effect on percent inhibition of HIV replication mediated by NK cells (p>0.05 for all comparisons, Wilcoxon matched pairs test). Addition of Abs to all 3 chemokines reduced NK-mediated HIV suppression. Comparisons of percent inhibition of HIV replication between NK+iCD4+neutralizing Abs to all 3 CC-chemokines and NK+iCD4 with either no Abs or antibodies to single CC-chemokines were significant for all comparisons except one at days 3 and 7 (p<0.05, Wilcoxon). The exception was the comparison of percent inhibition between NK+iCD4+neutralizing Abs to the 3 CC-chemokines and NK+iCD4 with no Abs (p = 0.23, Wilcoxon). None of the comparisons for percent inhibition at day 10 achieved statistical significance. These results indicate that iCD4 stimulated NK cell secretion of CC-chemokines contributes to inhibition on HIV replication.

### NK cells from individuals carrying protective *KIR/HLA* genotypes inhibit HIV replication more potently than those from *Bw6hmz*

**Figure 3:**
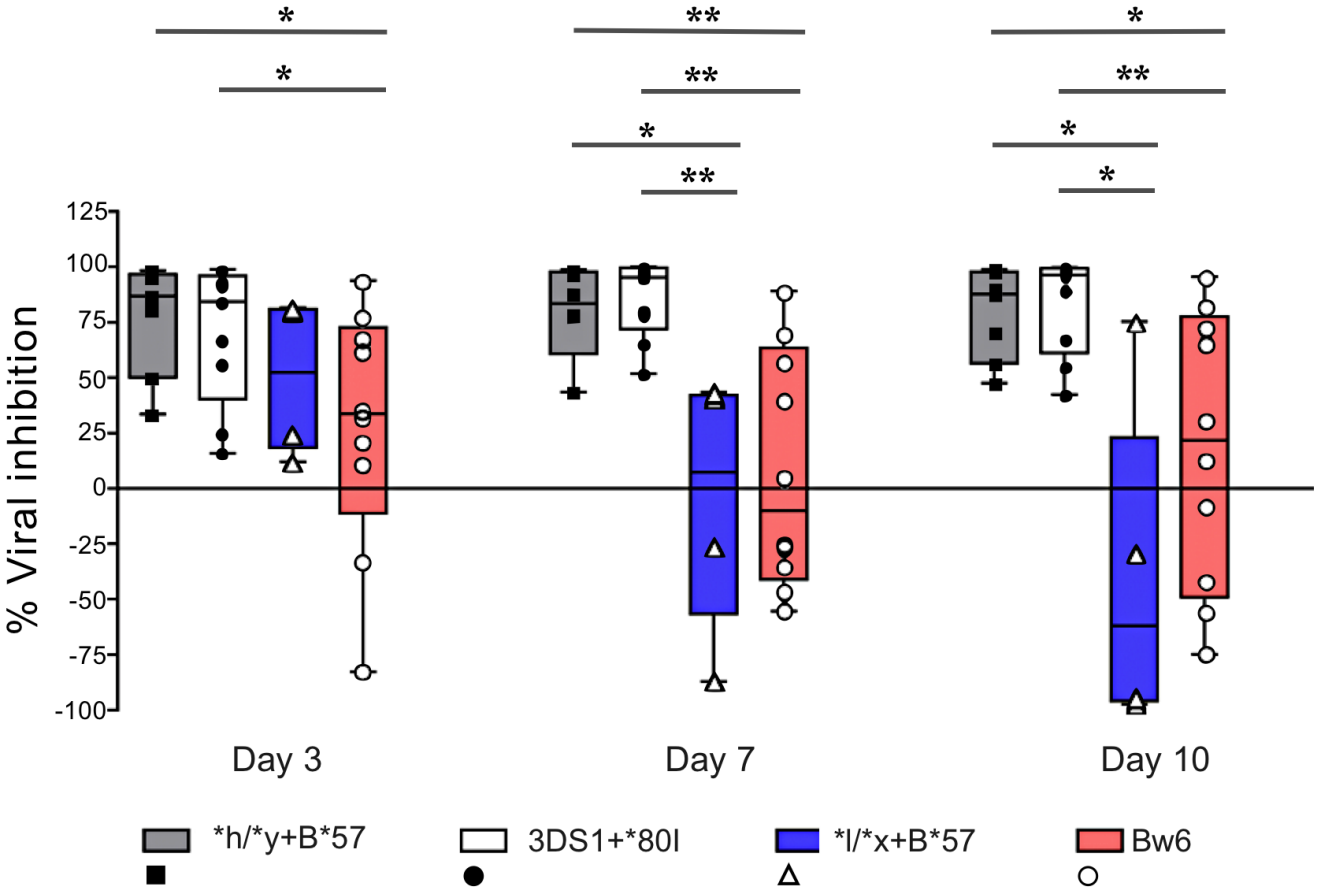
NK cells from subjects carrying *h/*y+B*57 and 3DS1+*80I suppress viral replication better than those from *Bw6hmz* and *l/*x+B*57 carriers. The box and whisker plots show the percent viral inhibition observed when NK cells from subjects positive for *h/*y+B*57 (n = 7) 3DS1+*80I (n = 9), *Bw6hmz* (n = 10) and *l/*x+B*57 (n = 4) are cultured with autologous HIV infected CD4 (iCD4) cells at a ratio of 10∶1 for up to 10 days. The line in each box represents the median value, the lower and upper limits of the boxes the 25% and 75% quartiles and the whiskers the minimum and maximum values for each group; each point is the percent viral inhibition value for a single individual. Lines linking groups indicate comparisons where medians were significantly different. “*” = p<0.05, “**” = p<0.01.

We next questioned whether NK cells from carriers of *h/*y+B*57, a genotype combination that confers protection from HIV disease progression, VL control and lowered infection risk, inhibits viral replication better than NK cells from *Bw6hmz* [7], [9]. Fig. 3 shows results for inhibition of HIV replication by NK cells from subjects positive for *h/*y+B*57 (n = 7), 3DS1+*80I (n = 9), *l/*x+B*57 (n = 4) and *Bw6hmz* (n = 11). In this experiment NK cells from 3DS1+*80I carriers are used as a positive control since Alter et al. had previously shown their capacity to inhibit HIV replication in autologous iCD4 cells [18]. NK cells from *h/*y+B*57 carriers inhibited HIV replication better than those from *Bw6hmz* and this was significant at all times tested (p = 0.01, 0.007, and 0.05 for days 3, 7, and 10, respectively, Mann-Whitney test). They also inhibited HIV replication better than those from *l/*x+B*57 carriers (p<0.05 for days 7 and 10). We confirmed that NK cells from 3DS1+*80I carriers inhibit HIV replication better than those from *Bw6hmz* and *l/*x+B*57 carriers (p<0.05 for all comparisons at days 7 and 10). NK cells from carriers of 3DS1+*80I and *h/*y+B*57 inhibit viral replication in autologous iCD4 cells with a similar potency at the times tested. We verified that these results are not due to a differential ability of HIV to replicate in CD4 cells from subjects carrying these 4 genotypes (Fig. S3). HIV p24 levels in culture supernatants of iCD4 cells from carriers of the 4 genotypes was equivalent at all times tested (p>0.05, Kruskal-Wallis test). Together these results show that NK cells from carriers of the *h/*y+B*57 genotype inhibit HIV replication in autologous CD4 cells better than those from *Bw6hmz* or carriers of the *l/*x+B*57 genotype.

### NK cells from individuals carrying protective *KIR/HLA* genotypes secrete higher levels of CC-chemokines than those from *Bw6hmz*

**Figure 4:**
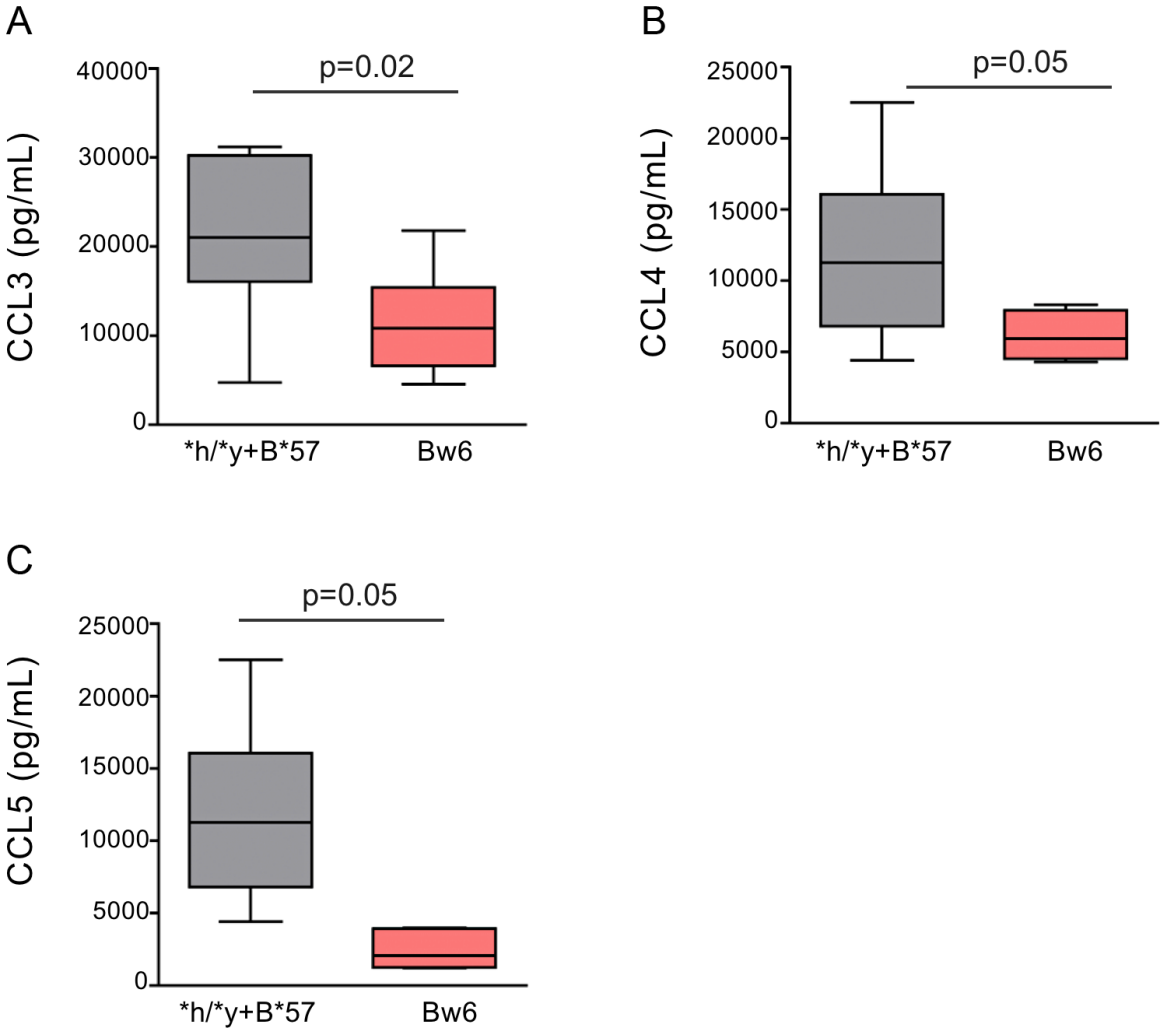
NK cells from subjects positive for *h/*y+B*57 secrete more CC-chemokines in response to autologous HIV infected CD4 (iCD4) cells than those from *Bw6hmz*. Box and whisker plots show the levels of CCL3 (A), CCL4 (B) and CCL5 (C) secreted over 3 days into the supernatant of cultures of NK cells and autologous iCD4 cells at a 10∶1 ratio from individuals positive for *h/*y+B*57 (n = 7) or from *Bw6hmz* (n = 10). The line in each box represents the median value, the lower and upper limits of the boxes the 25% and 75% quartiles and the whiskers the minimum and maximum values for each group. Lines linking groups indicate comparisons where medians were significantly different.

We next asked whether NK cells from individuals carrying protective *KIR/HLA* genotype combinations and *Bw6hmz* differed from each other in the amount of CC-chemokines they secreted upon stimulation with autologous iCD4 cells. We assessed the amount of CC-chemokines secreted over 3 days by NK cells from 7 *h/*y+B*57, 12 3DS1+*80I and 5 *l/*x+B*57 carriers and 10 *Bw6hmz*. Stimulated NK cells from *h/*y+B*57 and 3DS1 +*80I carriers secreted similar levels of CCL3, CCL4 and CCL5 to each other and more than those from *Bw6hmz* (Figs. 4, S4, S5 and Table S1). CC-chemokine secretion by stimulated NK cells from *l/*x+B*57 carriers was similar to that from *Bw6hmz* for CCL3 and CCL5 and higher than that from *Bw6hmz* for CCL4 (Fig. S5 and Table S1). In general, iCD4 stimulated NK cells from *h/*y+B*57 and 3DS1+*80I carriers secreted higher CC-chemokine levels compared to those from *l/*x+B*57 carriers, though several of these comparisons did not achieve statistical significance (Fig. S5 and Table S1).

**Figure 5:**
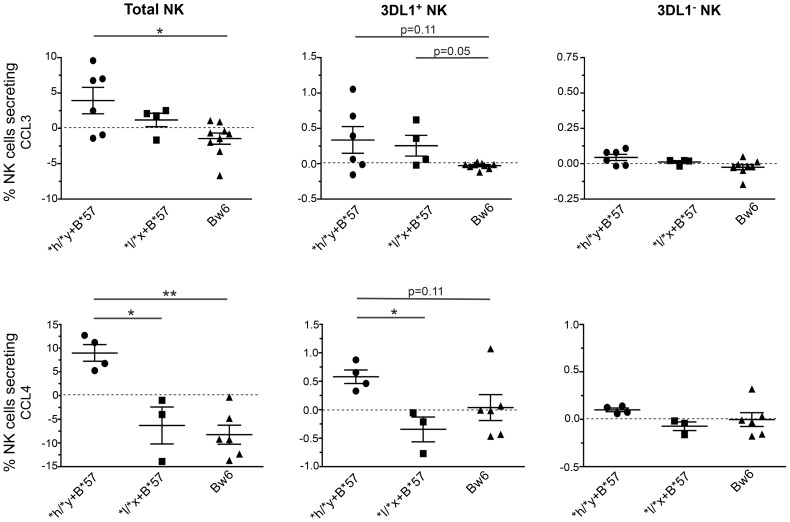
Percent of CCL3+ and CCL4+ NK cells and NK cell subsets following stimulation with autologous HIV infected CD4 (iCD4) cells. CD4 cells infected with HIV and cultured for 7 days were used to stimulate autologous NK cells for 24+ (upper panels) and CCL4+ (lower panels) total NK cells (left), KIR3DL1^+^ (middle) and KIR3DL1^−^ (right) NK cell subsets in subjects positive for *h/*y+B*57 (n = 7) *l/*x+B*57 (n = 4) and *Bw6hmz* (n = 9). Each point represents the value for a single individual, the line and error bars through each group show the mean and the standard error of the mean for each data set. Lines linking groups indicate between-group comparisons. “*” = a p-value<0.05, “**” = a p-value of <0.01.

We also stimulated NK cells overnight with autologous 7 day iCD4 and assessed intracellular CCL3, CCL4, IFN-γ and CD107a expression by total NK cells as well as by 3DL1^+^ and 3DL1^−^ NK cell subsets using the gating strategy shown in Fig. S6. Fig. 5 shows for CCL3 in the upper and CCL4 in the lower panels that a higher frequency of NK cells from *h/*y+B*57 carriers secrete these chemokines upon stimulations with autologous iCD4 than those from *Bw6hmz*. A similar but non-significant trend is when 3DL1^+^ NK cells are gated on that is absent in the 3DL1^−^ population (Fig. 5). We also compared the frequency of 3DL1^+^ and 3DL1^−^ cells within individuals secreting CCL3, CCL4 and IFN-γ and expressing CD107a (Figs. S7, S8, S9, S10). In general, a higher frequency of functional 3DL1^+^ than 3DL1^−^ NK cells was observed in *h/*y+B*57 carriers (p = 0.15, 0.02, 0.05 for CCL3, CCL4 and IFN-γ secretion, respectively), but not in *l/*x+B*57 carriers and *Bw6hmz*. It would have been desirable to compare the frequency of intracellular CCL3, CCL4 and IFN-γ positive cell in 3DL1^+^ *h versus *l allele expressing NK cell subsets following iCD4 stimulation of *l/*x+B*57 NK cells. Unfortunately, only 2 *l/*x+B*57 subjects carried both an *h and *l allele. The others were either homozygous for *l alleles or carried an *l and an *004 allele. The composition of the *l/*x+B*57 group precluded making firm conclusions regarding CC-chemokine or IFN-γ expression in these 3DL1^+^ NK subsets. Together, the intracellular cytokine staining results show that KIR/HLA genotype is a determinant of iCD4 stimulated NK cell functionality with regard to CC-chemokine secretion. The higher functionality of 3DL1^+^ NK cells in *h/*y+B*57 compared to *l/*x+B*57 carriers and *Bw6hmz* implicates this *KIR/HLA* combination in potent NK cell licensing for functional potential.

## Discussion

In this report we showed that NK cells cultured with autologous iCD4 cells limit the spread of HIV resulting in a lower frequency of HIV iCD4 cells and lower levels of viral replication compared to iCD4 cells cultured alone. Contact between NK and iCD4 cells stimulates NK cells to produce soluble factors, which suppress HIV replication in a non-contact dependent fashion. NK cells activated by autologous iCD4 cells in the presence of IL-2 secrete CC-chemokines at higher levels than when only IL-2 is present. CC-chemokine secretion is responsible, at least in part, for the inhibitory effect of NK cells on viral replication. *KIR/HLA* genotype influences the potency of inhibition of viral replication. We showed that NK cells from *h/*y+B*57 and 3DS1+*80I carriers, genotypes associated slower time to AIDS and VL control, inhibited HIV replication more potently than did those from *Bw6hmz* and carriers of the *l/*x+B*57genotype. NK cells, and in particular the 3DL1^+^ subset of NK cells, from carriers of the *h/*y+B*57 genotype secrete higher levels of CC-chemokines than those from *Bw6hmz* and *l/*x+B*57 subjects.

The superior control of HIV replication in autologous iCD4 cells by NK cells from carriers of *h/*y+B*57 versus those from *l/*x+B*57 and *Bw6hmz* subjects implicates NK cell education as a determinant of this anti-viral NK function. NK cell education is important for the development of self-tolerant NK cells and for endowing NK cells with the capacity to mediate cytokine/chemokine secretion and cytolysis upon encountering cells with reduced MHC-I cell surface expression such as occurs in the context of HIV infected targets [11], [21], [22]. The ligation of inhibitory NK receptors such as 3DL1 is required for NK education but the process is tuned by the set of signals received from all the NK cell surface activating and inhibitory receptors interacting with their ligands on neighboring target cells [23]–[25]. The stronger the inhibitory signals received during NK cell education the broader and more potent the effector functions that NK cells will have against appropriate targets [23]. The *h/*y+B*57 KIR/HLA combination appears to be a particularly potent one for NK cell education, since NK cells from *h/*y+B*57 carriers showed higher functionality when stimulated with HLA-null cells than those from carriers of 3DL1*h/*y genotypes co-carried with other *Bw4* or *80I alleles, 3DL1*l/*x genotypes co-carried with B*57 or those from *Bw6hmz* [10], [15]. The difference in functional potential between NK cells from carriers of *h/*y+B*57 versus those from *3DL1hmz* who carry other *Bw4* alleles may reflect differences in the impact of HLA-B*57 versus other Bw4 antigens in providing educational signals to NK cells during development. Transgenic mice expressing single MHC-I alleles have been used to show that MHC-I antigens differ in their impact on NK cell education [24]. The strength of the inhibitory input during education, as determined by the strength of the interaction between inhibitory NK receptors and their ligands, is directly related to the functional responsiveness of individual NK cells [23], [24]. Thus, it appears that B*57 differs from most other Bw4 molecules in the strength with which it interacts with 3DL1 to educate NK cells. NK cells from 3DL1*h/*y positive subjects express higher levels of 3DL1 inhibitory receptors than those from 3DL1*l/*x positive individuals [6] The observation that NK cells from *l/*x+B*57 carriers secrete less CC-chemokines and inhibit HIV replication more poorly than those from *h/*y+B*57 carriers may be related to less potent NK education due to lower levels of cell surface 3DL1 mediating lower inhibitory signals for NK cell education, even in the presence of the potent B*57 3DL1 ligand. A caveat to this interpretation is that while there is experimental evidence that B*57 binds 3DL1 it has not been demonstrated that the affinity of the interaction between these 2 molecules is greater than that between 3DL1 and other Bw4 molecules because different peptides influence 3DL1 Bw4 binding [26], [27]. In the presence of the same epitope and 3DL1 receptor HLA-Bw4*80T variants bind with about 60% of the affinity of B*57 [27].

The impact of *h/*y+B*57 on NK cell education and the relationship between NK education and NK cell responsiveness may underlie epidemiological findings that carriers of this genotype have a lower risk of HIV infection and in those who become infected have a slower time to AIDS and lower VL than carriers of other *3DL1 hmz Bw4* genotypes, including *l/*x+B*57 carriers [7], [9]. The influence of *h/*y+B*57 on NK cell education may also play a role in the superior ability of NK cells from carriers of this *KIR/HLA* genotype to inhibit viral replication in autologous HIV infected cells compared to those from *Bw6hmz*.

It is notable that the frequency of p24 positive CD4 cells in conditions where NK and iCD4 cells are in separate transwells is lower than that of iCD4 cells cultured alone but higher than that of iCD4 cells and NK cells cultured together. This implies that NK-CD4 cell contact contributes to NK cell activation and secretion of soluble factors that can inhibit HIV replication in a non-contact dependent manner. IL-2 by itself can also activate NK cells to secrete soluble factors such as CC-chemokines, though at lower levels than when iCD4 cells are also present. This may be why the percent of p24+ CD4 cells in conditions where iCD4 and NK cells are in separate transwell chambers is not as high as when iCD4 are cultured alone. It is not known whether these soluble factors are limited to CC-chemokines. Simultaneous neutralization of the CCL3, CCL4 and CCL5 restored HIV replication measured at 3 and 7 days of culture to levels that were significantly higher than when NK and iCD4 cells were co-cultured in the absence of CC-chemokine neutralization. Neutralization of all 3 CC-chemokines was not sufficient to reduce NK cell mediated inhibition of HIV replication at day 10 of culture. The reason for this is unclear but may be due to the continued production of chemokines over and above the amounts that anti-CC-chemokine Abs are able to neutralize. High inter-subject variability precludes making a clear determination as to whether CC-chemokine neutralization is sufficient to reverse NK cell mediated inhibition. It is possible that iCD4 stimulate NK cells to inhibit HIV replication by other mechanisms in addition to CC-chemokine secretion. These activities could target other stages of the HIV replication cycle and may or may not be dependent on contact between NK and iCD4 cells.

Previous studies have shown that NK cells secrete CC-chemokines following stimulation through CD16 cross-linking and co-culture with iCD4 cells in the presence of IL-2 [20]. Here we report for the first time that a KIR/HLA genotype combination that influences the potency of NK cell education also determines the level of CC-chemokines that NK cells secrete in response to autologous iCD4 cells. CC-chemokines can bind CCR5, the HIV co-receptor, and prevent HIV from interacting with this receptor thus reducing HIV entry [19], [20]. Transwell experiments implicate cell contact as a factor in NK cell stimulation leading to CC-chemokine secretion.

Pelak et al. reported that in carriers of 3DS1+*80I, the copy number of 3DL1 alleles influenced NK cell mediated inhibition of HIV replication in autologous iCD4 T cells [28]. Copy number variation (CNV) is common at the *3DL1/S1* locus. Screening for CNV at this locus revealed no duplications or deletions at this locus among subjects having the 4 genotypes focused on in this study. Therefore, CNV at the *3DL1/S1* locus can be excluded as a factor influencing the experimental findings reported here.

In summary, we show that NK cells from carriers of *h/*y+B*57 inhibit HIV viral replication in autologous iCD4 cells more effectively than those from *l/*x+B*57 carriers and *Bw6hmz*. The level of anti-viral function of NK cells from carriers of this genotype is likely related to NK cell education arising from B*57 interactions with high expression inhibitory 3DL1 receptors. Anti-viral function is mediated at least in part by CC-chemokine secretion levels able to block HIV entry into CD4 cell targets. The higher level of CC-chemokine secretion by NK cells from carriers of protective versus non-protective KIR/HLA genotypes may underlie their superior ability to inhibit HIV replication in infected targets.

## Materials and Methods

### Ethics statement

This study was conducted according to the principles expressed in the Declaration of Helsinki. It was approved by the Institutional Review Boards of the Comité d'Éthique de la Recheche du Centre Hospitalier de l'Université de Montréal and the Research Ethics Committee of the McGill University Health Centre - Montreal General Hospital. All subjects provided written informed consent for the collection of samples and subsequent analysis.

### Study population

We studied 47 HIV seronegative individuals, including 7 who were positive for *h/*y+B*57, 12 for 3DS1+*80I, 11 who were *3DL1hmz* and *Bw6hmz*, 4 who were *l/*x+B*57 positive and 13 with other *KIR/HLA* genotypes (Table 1). Informed consent was obtained from all study subjects, and the research conformed to all ethical guidelines of all the authors' institutions.

**Table 1:** Characteristics of study subjects.

ID	Age	Gender	Category	3DL/S1 Genotype	HLA Genotype					
					A		B		C	
1001	50	M	*h/*y+B57	3DL1_HMZ *h/*y_	A*01:01	A*26:01	B*38:01	B*57:01	C*06:02	C*12:03
1002	54	M	*h/*y+B57	3DL1_HMZ *h/*y_	A*02:22	A*03:01	B*44:02	B*57:01	C*05	C*06:02
1003	25	M	*h/*y+B57	3DL1_HMZ *h/*y_	A*01:01	A*02:01	B*38:01	B*57:01	C*06:02	C*12:03
1004	49	F	*h/*y+B57	3DL1_HMZ *h/*y_	A*02:01	A*02:01	B*57:01	B*57:01	C*03:03	C*07:01
1005	58	M	*h/*y+B57	3DL1_HMZ *h/*y_	A*24:02	A*25:01	B*37:01	B*57:01	C*06:02	C*06
1006	35	M	*h/*y+B57	3DL1_HMZ *h/*y_	A*01:01	A*03:01	B*14:02	B*57:01	C*06:02	C*08:02
1007	30	M	*h/*y+B57	3DL1_HMZ *h/*y_	A*01:01	A*02:01	B*15:01	B*57:01	C*05	C*06:02
2008	45	M	3DS1+80I	HTZ_high_	A*02:01	A*26:01	B*38:01	B*44:02	C*05	C*16:04
2009	47	M	3DS1+80I	HTZ_high_	A*01:01	A*23:01	B*44:03	B*57:01	C*04:01	C*06:02
2010	52	M	3DS1+80I	HTZ_null (004)_	A*02:01	A*02:01	B*44:02	B*49:01	C*05	C*07:01
2011	60	M	3DS1+80I	HTZ_high_	A*02:01	A*03:01	B*14:02	B*51:01	C*02:02	C*08:02
2012	35	F	3DS1+80I	HTZ_high_	A*11:01	A*26:01	B*07:02	B*57:01	C*06:02	C*07:02
2013	45	F	3DS1+80I	HTZ_high_	A*01:01	A*02:01	B*38:01	B*57:01	C*06:02	C*12
2014	44	M	3DS1+80I	HTZ_high_	A*01:01	A*24:02	B*35:01	B*52:01	C*04:01	C*12:02
2015	28	M	3DS1+80I	HTZ_high_	A*02:01	A*02:01	B*44	B*51	C*05:01/02	C*15:02
2016	51	M	3DS1+80I	HTZ_high_	A*03:01	A*11:01	B*40:02	B*57:01	C*02:02	C*04:01
2017	31	M	3DS1+80I	HTZ_high_	A*03:01	A*32:01	B*13:02	B*53:01	C*04:01	C*06:02
2018	42	M	3DS1+80I	HTZ_null (004)_	A*01:01	A*26:01	B*52:01	B*55:01	C*03:03	C*12:02
2019	44	F	3DS1+80I	3DS1_HMZ_	A*01:01	A*01:01	B*08:01	B*57:01	C*07:01	C*07:01
3020	44	M	Bw6	3DL1_HMZ *h/*y_	A*02:01	A*25:01	B*18:01	B*55:01	C*03:03	C*12:03
3021	38	M	Bw6	3DL1_HMZ *h/*y_	A*03:01	A*11:01	B*07:02	B*35:01	C*04:01	C*07
3022	55	M	Bw6	3DL1_HMZ *l/*x_	A*01:01	A*02:01	B*08:01	B*08:01	C*07	C*07
3023	42	F	Bw6	HTZ_high_	A*02:01	A*03:01	B*15:01	B*07:02	C*03	C*07
3024	49	M	Bw6	3DL1_HMZ *h/*y_	A*02:01	A*32:01	B*18:01	B*50:01	C*06:02	C*07:01
3025	48	M	Bw6	3DL1_HMZ *h/*y_	A*02:01	A*03:01	B*07:02	B*40:01	C*03:02	C*07
3026	48	M	Bw6	3DL1_HMZ *h/*y_	A*01:01	A*24:03	B*07:02	B*07:02	C*07	C*07
3027	49	M	Bw6	HTZ_high_	A*31:01	A*68:01	B*40:01	B*40:01	C*03:04	C*07:01
3028	44	M	Bw6	HTZ_high_	A*02:01	A*02:01	B*35:01	B*35:01	C*05	C*05
3029	45	M	Bw6	3DL1_HMZ *l/*x_	A*02:01	A*33:03	B*15:01	B*35:08	C*03:03	C*04:01
3030	45	M	Bw6	3DL1_HMZ *h/*y_	A*02:01	A*02:01	B*07:02	B*08:01	C*07	C*07
4031	36	F	*l/*x+B57	3DL1_HMZ *l/*x_	A*02:01	A*02:01	B*07:02	B*57:01	C*05	C*06:02
4032	68	M	*l/*x+B57	3DL1_HMZ *l/*x_	A*24:02	A*26:01	B*15:01	B*57:01	C*05	C*06:02
4033	44	M	*l/*x+B57	3DL1_HMZ *l/*x_	A*02:01	A*02:01	B*15:01	B*57:01	C*03:04	C*06:02
4034	33	F	*l/*x+B57	3DL1_HMZ *l/*x_	A*02:02	A*30:02	B*53:01	B*57:03	C*04:01	C*18
5035	59	F	3DS1_HMZ_+_non_80I	3DS1_HMZ_	A*01:01	A*02:01	B*08:01	B27:05	C*02:02	C*07:01
5036	36	M	3DS1_HMZ_+_non_80I	3DS1_HMZ_	A*02:01	A*03:01	B*07:02	B*56:01	C*01:02	C*07
5037	42	M	3DS1_HMZ_+_non_80I	3DS1_HMZ_	A*03:01	A*30:01	B*27	B*35:01	C*01	C*04:01
5038	49	M	3DS1_HMZ_+_non_80I	3DS1_HMZ_	A*02:01	A*23:01	B*07:02	B*35:03	C*12:03	C*12:03
5039	22	M	3DS1_HMZ_+_non_80I	3DS1_HMZ_	A*01:01	A*02:03	B*37:01	B*46:01	C*01:02	C*06:02
5040	52	M	3DS1_HMZ_+_non_80I	3DS1_HMZ_	A*02	A*03	B*08	B*44		
5041	53	M	*80T	3DL1_HMZ *l/*x_	A*02:01	A*03:01	B*07:02	B*27:05	C*01:02	C*07:02
5042	46	M	*80I not B*57	3DL1_HMZ *h/*y_	A*01:01	A*31:01	B*49:01	B*49:01	C*07:01	C*07:01
5043	45	M	*80I not B*57	3DL1_HMZ *h/*y_	A*31:01	A*68:01	B*27	B*51:01	C*02:02	C*12:03
5044	43	M	*80I not B*57	3DL1_HMZ *l/*x_	A*11:01	A*11:01	B*27:05	B*53:01	C*02:02	C*04:01
5045	45	F	*80I not B*57	3DS1_HMZ_	A*02:01	A*24:02	B*38:01	B*78:01	C*07:02	C*07:02
5046	49	M	*80I not B*57	3DL1_HMZ *l/*x_	A*24:02	A*29:02	B*07:02	B*35:01	C*04:04	C*07:02
5047	29	M	*80I not B*57	3DL1_HMZ *h/*y_	A*01:01	A*03:01	B*44:03	B*49:01	C*07:01	C*16:01

### Genotyping

All subjects were typed for MHC-I alleles by sequence based typing using kits from Atria Genetics, Inc. (South San Francisco, CA). Assign 3.5+ software was used to interpret sequence information for allele assignment (Conexio Genetics, Perth, Australia). *KIR3DL1/S1* generic genotyping was performed by PCR using 2 pairs of primers specific for either *3DL1* or *3DS1* alleles as previously described [29]. *3DL1* allotyping was done by sequencing *3DL1* exons as previously described [9]. Single nucleotide polymorphisms (SNP) corresponding to the *3DL1* alleles were identified by aligning the sequenced DNA to a reference consensus sequence consisting of *3DL1* cDNA sequences. The *h/*y genotype refers to a *3DL1* homozygous genotype with no *l alleles. *Bw6hmz* lacked *Bw4* alleles at the *HLA-A* and *-B* loci.

### Cell purification

Peripheral blood mononuclear cells (PBMC) were isolated from blood by density gradient centrifugation (Ficoll-Paque; Pharmacia, Uppsala, Sweden) and cryopreserved in 10% dimethyl sulfoxide (DMSO; Sigma-Aldrich, St. Louis, MO) with 90% fetal bovine serum (FBS; Wisent, Inc. St. Bruno, QC, Canada). CD4 T cells were isolated from thawed PBMC by positive selection using immunomagnetic beads (STEMCELL Technologies, Inc. Vancouver, BC, Canada). The purity of the CD4 cell population was verified by flow cytometry (average 95.3%). NK cells were isolated from thawed PBMC by negative selection (STEMCELL Technologies, Inc.) and yielded an average purity of 97.2%.

### Inhibition of viral replication assay

Purified CD4 cells (10^6^/ml) were stimulated with 1 ug/ml PHA-P (MP Biomedicals, Santa Ana, CA) and 100 international units (IU)/ml of IL-2 (Chiron Corp., Emeryville, CA) overnight in RPMI medium containing 2 mM L-glutamine, 100 IU/ml Penicillin, 100 µg/ml Streptomycin (cRPMI) (all from Wisent) supplemented with 10% FBS, (Wisent, [R10]) at 37°C in a 5% CO_2_ humidified incubator. Stimulated CD4 cells were then washed three times with cRPMI supplemented with 2% FBS (R2), and cultured in R10 with 100 IU IL-2 for 3 days. On day 4, CD4 cells were infected at a multiplicity of infection of 0.01 with HIV-1_JR CSF_ in R10 for 4 hrs and washed three times with R2. Equal numbers (3.0 to 4.0×10^4^) of these iCD4 cells were plated at NK∶iCD4 ratios of 10∶1, 1∶1 or alone for 10 days in 300 ul of R10; 100 IU/ml IL-2. Supernatants were collected by removing supernatants and replenishing wells with 300 ul of fresh R10; 100 IU/ml IL-2 on days 3, 7 and 10 for assessment of p24 levels and on days 1, 2 and 3 for assessment of CC-chemokine levels.

For some experiments CD4 cells were collected on days 3, 7 and 10 for intracellular Gag p24 staining. Cells were stained with an Aqua amine reactive fluorescent dye (Invitrogen, Burlington, ON, Canada) to identify viable cells. Cell surface staining with anti-CD3 APC-eFluor 780 (eBioscience, San Diego, CA) and anti-CD4 PE (BD Biosciences, Mississauga, ON, Canada) were used to detect CD4 T cells. After fixation and permeabilization intracellular HIV Gag p24 positive cells were detected using the mAb KC57 (Beckman-Coulter, Mississauga, ON, Canada). Acquisition was done on a BD FACSCanto II flow cytometer (BD Biosciences, San Jose, CA). Between 50,000 and 200,000 events were acquired per sample. Flow cytometry results were analyzed with Flowjo software Mac 9.4 (Treestar, Ashland, OR). The gating strategy used for intracellular p24 positive cells is shown in Fig. S2.

To ascertain the requirement for NK-CD4 cell contact for NK cell–mediated inhibition of HIV replication, autologous NK cells were physically separated from iCD4 cells in transwell plates (Corning, Tewksbury MA). iCD4 cells (10^5^/well) were cultured in the upper chamber with either 10^6^ NK cells alone or 10^6^ NK cells with 10^5^ iCD4 T cells in the lower chamber. Cells in wells containing iCD4 cells were collected on days 3, 7 and 10 to quantitate the frequency of HIV Gag p24 positive CD4 cells.

#### NK cell stimulation

iCD4 or uninfected CD4 cells were prepared as above and cultured in R10; 100 IU/ml IL-2. Purified NK cells, isolated as described above were plated at a 10∶1 ratio with either iCD4 or CD4 cells for 24 hrs in R10; 100 IU/ml IL-2. Brefeldin (6 ug/ml, Sigma-Aldrich) and monensin (5 ug/ml, Golgi Stop, BD Biosciences) were added 5 hrs before the end of the culture period. Cells were stained for viability using an amine reactive dye (Invitrogen) and their Fc receptors were blocked using the TruStain FcX reagent (BioLegend, San Diego, CA) according to manufacturer's directions. Cells were then stained for surface markers with CD3-BV605 (OKT3), CD56-BV711 (NCAM), 3DL1-BV421 (DX9) (all from BioLegend) and anti-CD107a-PE-CF594 (BD), for 30 min. Samples were washed with phosphate buffered saline (PBS) containing 1% FBS (Wisent), fixed and permeabilized using a cell fixation and permeabilization kit (Invitrogen) and stained for intracellular cytokines/chemokines using anti-CCL3-APC (93342), anti-IFN-γ-Alexa700 (B27) (BD) and anti-CCL4-FITC (24006) (R&D Systems, Minneapolis, MN), for 15 min. After washing, samples were fixed with a solution of 1% paraformaldehyde (Fisher Scientific, Ottawa, ON, Canada) and kept in the dark at 4°C until acquisition.

Between 400,000 and 1,500,000 total events were acquired for each sample on an LSRFortessa flow cytometer (BD).The gating strategy used to analyze the functional profile of NK cells stimulated with iCD4 or uninfected CD4 cells is shown in Fig. S6. For all analyses, NK cells were defined as CD3^−^CD56^+^. The percent of CCL3, CCL4, IFN-γ and CD107a positive total, 3DL1^+^ and 3DL1^−^ NK cells was determined. Flow cytometry analysis for NK cell activation following stimulation was performed using FlowJo software. The results for the percent of functional NK cells stimulated with iCD4 reported were background corrected for NK cells stimulated with uninfected CD4 cells. Statistical analyses were performed using background corrected results.

#### HIV p24 ELISA

An Ab-sandwich ELISA was used to detect HIV Gag p24 in culture supernatants as described previously [30]. Briefly, 96-well ELISA plates were coated with anti-p24 Ab clone 183 H12-5C at 2.5 ug/ml overnight at RT. Culture supernatants (100 ul/well) were added for 1 hr at 37°C. The following sequential additions were made with 3 washes between steps using PBS; 0.05% Tween 20 (Sigma-Aldrich): 1) biotinylated anti-p24 Ab clone 31-90-25 at 0.5 ug/ml for 1 hr at 37°C, 2) 0.067 ug/ml horseradish peroxidase conjugated-steptavidin (Fitzgerald Industries International, Acton, MA) for 30 min at RT and 3) 3, 3′, 5, 5′-tetramethylbenzidine (TMB) substrate (Sigma-Aldrich) for 20 min at RT. Color development was stopped with 50 ul of 1 M H_2_SO_4_. ELISA plates were read at OD_450_ on an ELISA plate reader (PerkinElmer, Montreal, QC, Canada). The p24 concentration in test supernatants was determined by comparison with a p24 standard curve included on each plate. Percent viral inhibition was calculated using the equation [(p24 levels in iCD4 wells – p24 levels in NK+iCD4 wells)/(p24 levels in iCD4 wells) *100].

#### CC-chemokine ELISA

Culture supernatant levels of CC-chemokines was measured using Ab sandwich ELISAs detecting CCL3, CCL4 and CCL5 (R&D Systems) according to directions provided by the manufacturer.

#### Statistical analysis

Statistical analyses and graphical presentations were performed using GraphPad InStat 3.05 and GraphPad Prism 5. Mann-Whitney and Kruskal-Wallis tests with Dunn's post tests were used to compare the significance of mean differences between 2 and more than 2 groups, respectively. Wilcoxon matched pairs and Friedman tests were used to compare the effect of a condition on 2 or more than 2 matched groups, respectively. P-values less than 0.05 were considered significant.

## Supporting Information

Figure S1 **NK cells inhibit HIV replication in autologous HIV infected CD4 (iCD4) cells better at higher NK∶iCD4 cell ratios.** The scatter plots show percent inhibition in p24 Gag production in the presence versus the absence of NK cells at NK∶iCD4 cell ratios of 10∶1 and 1∶1 for days 3, 7 and 10 of culture. NK cells from individuals with the following *KIR/HLA* genotypes were used for this figure: *h/*y+B*57(n = 3), 3DS1+*80I (n = 2), *Bw6hmz* (n = 4) and other *KIR/HLA* (n = 8). Each point represents a separate individual. The lines and error bars through the scatter plots show the mean and standard error of the mean for that group. Lines linking groups indicate comparisons where means were significantly different. Friedman tests were used to compare the mean values at different time points for the same NK∶iCD4 cell ratios. Wilcoxon matched pairs tests were used to compare different NK∶iCD4 cell ratios at the same time point. “*” = p<0.05, “**” = p<0.01.
(TIFF)

Figure S2 **Gating strategy for detection of infected CD4 (iCD4) cells.** iCD4 cells co-cultured or not with autologous NK cells were surface stained with anti-CD3 and anti-CD4 antibodies. Cells were then permeabilized and stained for intracellular HIV p24 antigen and Aqua amine reactive dye to distinguish viable and non-viable cells. (A) Live CD3 positive cells were gated on from the lymphocytic singlet population. (B) The percentage of CD3 positive cells that stained for HIV p24 is shown in the boxed area. SSC = side scatter, FSC = forward scatter.
(TIFF)

Figure S3 **Infected CD4 (iCD4) cells from individuals carrying various** ***KIR/HLA*** **genotypes replicate HIV to similar levels.** The line graph depicts the mean change in log_10_ p24 levels secreted by iCD4 cells. Results were generated using iCD4 from subjects positive for *h/*y+B*57 (n = 7), 3DS1+*80I (n = 9), *Bw6hmz* (n = 10) and *l/*x+B*57 (n = 4).
(TIFF)

Figure S4 **NK cells from** 3DS1+*80I **carriers secrete more CC-chemokines in response to autologous HIV infected CD4 (iCD4) cells than those from** ***Bw6hmz*** **.** Box and whisker plots show levels of CCL3 (A), CCL4 (B) and CCL5 (C) secreted over 3 days into the supernatant of cultures of NK cells and autologous iCD4 cells at a 10∶1 ratio from individuals positive for 3DS1+*80I (n = 12) or from *Bw6hmz* (n = 10). The line in each box represents the median value, the lower and upper limits of the boxes the 25% and 75% quartiles and the whiskers the minimum and maximum values for each group. P-values are shown over lines linking groups being compared.
(TIFF)

Figure S5 **Secretion levels of CC-chemokines from NK cells responding to stimulation with autologous HIV infected CD4 (iCD4) categorized by** ***KIR/HLA*** **genotype.** Box and whisker plots show levels of CCL3 (A), CCL4 (B) and CCL5 (C) secreted over 3 days into the supernatant of cultures of NK cells and autologous iCD4 cells at a 10∶1 ratio from individuals positive for *h/*y+B*57 (n = 7) 3DS1+*80I (n = 12) *Bw6hmz* (n = 10) and *l/*x+B*57 (n = 4). The line in each box represents the median value, the lower and upper limits of the boxes the 25% and 75% quartiles and the whiskers the minimum and maximum values for each group. P-values are shown over lines linking groups being compared.
(TIFF)

Figure S6 **Gating strategy used to assess the percent of functional NK cells stimulated with autologous infected CD4 (iCD4) cells.** (A) We used FSC-A and SSC-A to gate on lymphocytes and FSC-A and FSC-H to gate on single cell events from co-cultures of NK cells with autologous CD4 or iCD4 cells. Only live CD3^−^CD56^+^ NK cells were included in this analysis. The representative subject shown in this figure carries a KIR3DL1 *l/*x genotype with 1 high and 1 low *KIR3DL1* allele. (B) Functional gates were set using unstimulated PBMCs that were gated on the KIR3DL1^+^ NK cell population using the gating strategy shown in panel A. The percent of CCL3, CCL4, IFN-γ and CD107a positive cells was determined for conditions in which NK and CD4 cells were cultured with IL-2 (negative control) and NK cells and iCD4 cells were cultured with IL-2. NK responses to HIV iCD4 were background subtracted for responses to uninfected CD4 cells. FCS-A = forward scatter area; SSC-A = side scatter area; FSC-H = forward scatter height; PBMC = peripheral blood mononuclear cells.
(TIFF)

Figure S7 **Secretion of CCL3 from KIR3DL1^+^ (3DL1^+^) and KIR3DL1^−^ (3DL1^−^) NK subsets.** Paired scatter plots show the percent of 3DL1^+^ and 3DL1^−^ NK cells secreting CCL3 following stimulation with autologous infected CD4 (iCD4) cells. Shown are results for all individuals tested (upper left panel) and for subjects positive for *h/*y+B*57 (n = 6, upper right) *l/*x+B*57 (n = 4, lower left) and *Bw6hmz* (n = 4, lower right). The significance of between group differences in the percent of CCL3 secreting cells was tested using a Wilcoxon matched pairs test. P-values for between group comparisons are shown.
(TIFF)

Figure S8 **Secretion of CCL4 from KIR3DL1^+^ (3DL1^+^) and KIR3DL1^−^ (3DL1^−^) NK subsets.** Paired scatter plots show the percent of 3DL1^+^ and 3DL1^−^ NK cells secreting CCL4 following stimulation with autologous infected CD4 (iCD4) cells. Shown are results for all individuals tested (upper left panel) and for subjects positive for *h/*y+B*57 (n = 4, upper right) *l/*x+B*57 (n = 3, lower left) and *Bw6hmz* (n = 4, lower right). The significance of between group differences in the percent of CCL4 secreting cells was tested using a Wilcoxon matched pairs test. P-values for between group comparisons are shown.
(TIFF)

Figure S9 **Secretion of IFN-γ from KIR3DL1^+^ (3DL1^+^) and KIR3DL1^−^ (3DL1^−^) NK subsets.** Paired scatter plots show the percent of 3DL1^+^ and 3DL1^−^ NK cells secreting IFN-γ following stimulation with autologous infected CD4 (iCD4) cells. Shown are results for all individuals tested (upper left panel) and for subjects positive for *h/*y+B*57 (n = 6, upper right) *l/*x+B*57 (n = 4, lower left) and *Bw6hmz* (n = 4, lower right). The significance of between group differences in the percent of IFN-γ secreting cells was tested using a Wilcoxon matched pairs test. P-values for between group comparisons are shown.
(TIFF)

Figure S10 **Expression of CD107a in KIR3DL1^+^ (3DL1^+^) and KIR3DL1^−^ (3DL1^−^) NK subsets.** Paired scatter plots show the percent of 3DL1^+^ and 3DL1^−^ NK cells expressing CD107a following stimulation with autologous infected CD4 (iCD4) cells. Shown are results for all individuals tested (upper left panel) and for subjects positive for *h/*y+B*57 (n = 6, upper right) *l/*x+B*57 (n = 4, lower left) and *Bw6hmz* (n = 4, lower right). The significance of between group differences in the percent of CD107a expressing cells was tested using a Wilcoxon matched pairs test. P-values for between group comparisons are shown.
(TIFF)

Table S1 **P-values for pair-wise comparisons of CC-chemokine secretion levels by infected CD4 (iCD4) cells stimulated NK cells from individuals categorized by** ***KIR/HLA*** **genotype.** The significance of between group comparisons was assessed using Mann-Whitney tests.
(DOC)

## References

[1] Stetson DB, Mohrs M, Reinhardt RL, Baron JL, Wang ZE, et al (2003) Constitutive cytokine mRNAs mark natural killer (NK) and NK T cells poised for rapid effector function. J Exp Med 198: 1069–1076. 14530376 10.1084/jem.20030630 PMC2194220

[2] Lanier LL (2005) NK cell recognition. Annu Rev Immunol 23:225–74.: 225–274. 15771571 10.1146/annurev.immunol.23.021704.115526

[3] Bashirova AA, Thomas R, Carrington M (2011) HLA/KIR restraint of HIV: surviving the fittest. Annu Rev Immunol 29: 295–317 10.1146/annurev-immunol-031210-101332 [doi]. 21219175 10.1146/annurev-immunol-031210-101332 PMC3725604

[4] Pando MJ, Gardiner CM, Gleimer M, McQueen KL, Parham P (2003) The protein made from a common allele of KIR3DL1 (3DL1*004) is poorly expressed at cell surfaces due to substitution at positions 86 in Ig domain 0 and 182 in Ig domain 1. J Immunol 171: 6640–6649. 14662867 10.4049/jimmunol.171.12.6640

[5] Taner SB, Pando MJ, Roberts A, Schellekens J, Marsh SG, et al (2011) Interactions of NK cell receptor KIR3DL1*004 with chaperones and conformation-specific antibody reveal a functional folded state as well as predominant intracellular retention. J Immunol 186: 62–72 jimmunol.0903657 [pii]; 10.4049/jimmunol.0903657 [doi]. 21115737 10.4049/jimmunol.0903657 PMC3129036

[6] Yawata M, Yawata N, Draghi M, Little AM, Partheniou F, et al (2006) Roles for HLA and KIR polymorphisms in natural killer cell repertoire selection and modulation of effector function. J Exp Med 203: 633–645. 16533882 10.1084/jem.20051884 PMC2118260

[7] Martin MP, Qi Y, Gao X, Yamada E, Martin JN, et al (2007) Innate partnership of HLA-B and KIR3DL1 subtypes against HIV-1. Nat Genet 39: 733–740. 17496894 10.1038/ng2035 PMC4135476

[8] Wan AM, Ennis P, Parham P, Holmes N (1986) The primary structure of HLA-A32 suggests a region involved in formation of the Bw4/Bw6 epitopes. J Immunol 137: 3671–3674. 2431040

[9] Boulet S, Kleyman M, Kim JY, Kamya P, Sharafi S, et al (2008) A combined genotype of KIR3DL1 high expressing alleles and HLA-B*57 is associated with a reduced risk of HIV infection. AIDS 22: 1487–1491. 18614872 10.1097/QAD.0b013e3282ffde7e

[10] Kamya P, Boulet S, Tsoukas CM, Routy JP, Thomas R, et al (2011) Receptor-ligand requirements for increased NK cell poly-functional potential in *h/*y+B57 HIV-1 infected Slow progressors. J Virol JVI.02652-10 [pii]; 10.1128/JVI.02652-10 [doi]. 10.1128/JVI.02652-10 PMC3126301 21471235

[11] Kim S, Sunwoo JB, Yang L, Choi T, Song YJ, et al (2008) HLA alleles determine differences in human natural killer cell responsiveness and potency. Proc Natl Acad Sci U S A 105: 3053–3058 0712229105 [pii]; 10.1073/pnas.0712229105 [doi]. 18287063 10.1073/pnas.0712229105 PMC2268583

[12] Gumperz JE, Litwin V, Phillips JH, Lanier LL, Parham P (1995) The Bw4 public epitope of HLA-B molecules confers reactivity with natural killer cell clones that express NKB1, a putative HLA receptor. J Exp Med 181: 1133–1144. 7532677 10.1084/jem.181.3.1133 PMC2191933

[13] Kim S, Poursine-Laurent J, Truscott SM, Lybarger L, Song YJ, et al (2005) Licensing of natural killer cells by host major histocompatibility complex class I molecules. Nature 436: 709–713 nature03847 [pii];10.1038/nature03847 [doi]. 16079848 10.1038/nature03847

[14] Anfossi N, Andre P, Guia S, Falk CS, Roetynck S, et al (2006) Human NK cell education by inhibitory receptors for MHC class I. Immunity 25: 331–342. 16901727 10.1016/j.immuni.2006.06.013

[15] Boulet S, Song R, Kamya P, Bruneau J, Shoukry NH, et al (2010) HIV protective KIR3DL1 and HLA-B genotypes influence NK cell function following stimulation with HLA-devoid cells. J Immunol 184: 2057–2064 jimmunol.0902621 [pii];10.4049/jimmunol.0902621 [doi]. 20061407 10.4049/jimmunol.0902621

[16] Martin MP, Gao X, Lee JH, Nelson GW, Detels R, et al (2002) Epistatic interaction between KIR3DS1 and HLA-B delays the progression to AIDS. Nat Genet 31: 429–434. 12134147 10.1038/ng934

[17] Qi Y, Martin MP, Gao X, Jacobson L, Goedert JJ, et al (2006) KIR/HLA Pleiotropism: Protection against Both HIV and Opportunistic Infections. PLoS Pathog 2: e79. 16933987 10.1371/journal.ppat.0020079 PMC1550271

[18] Alter G, Martin MP, Teigen N, Carr WH, Suscovich TJ, et al (2007) Differential natural killer cell-mediated inhibition of HIV-1 replication based on distinct KIR/HLA subtypes. J Exp Med 204: 3027–3036. 18025129 10.1084/jem.20070695 PMC2118524

[19] Cocchi F, DeVico AL, Garzino-Demo A, Arya SK, Gallo RC, et al (1995) Identification of RANTES, MIP-1 alpha, and MIP-1 beta as the major HIV-suppressive factors produced by CD8+ T cells. Science 270: 1811–1815. 8525373 10.1126/science.270.5243.1811

[20] Oliva A, Kinter AL, Vaccarezza M, Rubbert A, Catanzaro A, et al (1998) Natural killer cells from human immunodeficiency virus (HIV)-infected individuals are an important source of CC-chemokines and suppress HIV-1 entry and replication in vitro. J Clin Invest 102: 223–231 10.1172/JCI2323 [doi]. 9649576 10.1172/JCI2323 PMC509084

[21] Collins KL, Chen BK, Kalams SA, Walker BD, Baltimore D (1998) HIV-1 Nef protein protects infected primary cells against killing by cytotoxic T lymphocytes. Nature 391: 397–401. 9450757 10.1038/34929

[22] Cohen GB, Gandhi RT, Davis DM, Mandelboim O, Chen BK, et al (1999) The selective downregulation of class I major histocompatibility complex proteins by HIV-1 protects HIV-infected cells from NK cells. Immunity 10: 661–671. 10403641 10.1016/s1074-7613(00)80065-5

[23] Brodin P, Lakshmikanth T, Johansson S, Karre K, Hoglund P (2009) The strength of inhibitory input during education quantitatively tunes the functional responsiveness of individual natural killer cells. Blood 113: 2434–2441 blood-2008-05-156836 [pii];10.1182/blood-2008-05-156836 [doi]. 18974374 10.1182/blood-2008-05-156836

[24] Johansson S, Johansson M, Rosmaraki E, Vahlne G, Mehr R, et al (2005) Natural killer cell education in mice with single or multiple major histocompatibility complex class I molecules. J Exp Med 201: 1145–1155 jem.20050167 [pii];10.1084/jem.20050167 [doi]. 15809355 10.1084/jem.20050167 PMC2213126

[25] Fauriat C, Ivarsson MA, Ljunggren HG, Malmberg KJ, Michaelsson J (2010) Education of human natural killer cells by activating killer cell immunoglobulin-like receptors. Blood 115: 1166–1174 blood-2009-09-245746 [pii];10.1182/blood-2009-09-245746 [doi]. 19903900 10.1182/blood-2009-09-245746

[26] Fadda L, O'Connor GM, Kumar S, Piechocka-Trocha A, Gardiner CM, et al (2011) Common HIV-1 peptide variants mediate differential binding of KIR3DL1 to HLA-Bw4 molecules. J Virol 85: 5970–5974 JVI.00412-11 [pii];10.1128/JVI.00412-11 [doi]. 21471246 10.1128/JVI.00412-11 PMC3126328

[27] Vivian JP, Duncan RC, Berry R, O'Connor GM, Reid HH, et al (2011) Killer cell immunoglobulin-like receptor 3DL1-mediated recognition of human leukocyte antigen B. Nature 479: 401–405 nature10517 [pii];10.1038/nature10517 [doi]. 22020283 10.1038/nature10517 PMC3723390

[28] Pelak K, Need AC, Fellay J, Shianna KV, Feng S, et al (2011) Copy number variation of KIR genes influences HIV-1 control. PLoS Biol 9: e1001208 10.1371/journal.pbio.1001208 [doi]; PBIOLOGY-D-11-02515 [pii]. 22140359 10.1371/journal.pbio.1001208 PMC3226550

[29] Boulet S, Sharafi S, Simic N, Bruneau J, Routy JP, et al (2008) Increased proportion of KIR3DS1 homozygotes in HIV-exposed uninfected individuals. AIDS 22: 595–599. 18317000 10.1097/QAD.0b013e3282f56b23

[30] Bounou S, Leclerc JE, Tremblay MJ (2002) Presence of host ICAM-1 in laboratory and clinical strains of human immunodeficiency virus type 1 increases virus infectivity and CD4(+)-T-cell depletion in human lymphoid tissue, a major site of replication in vivo. J Virol 76: 1004–1014. 11773376 10.1128/JVI.76.3.1004-1014.2002 PMC135853

