# Supplementary figures and images for: HIV Protective KIR3DL1/S1-HLA-B Genotypes Influence NK Cell-Mediated Inhibition of HIV Replication in Autologous CD4 Targets
Source: PLoS Pathog. 2014 Jan 16;10(1):e1003867. doi: 10.1371/journal.ppat.1003867 (PMC3894215; doi:10.1371/journal.ppat.1003867)

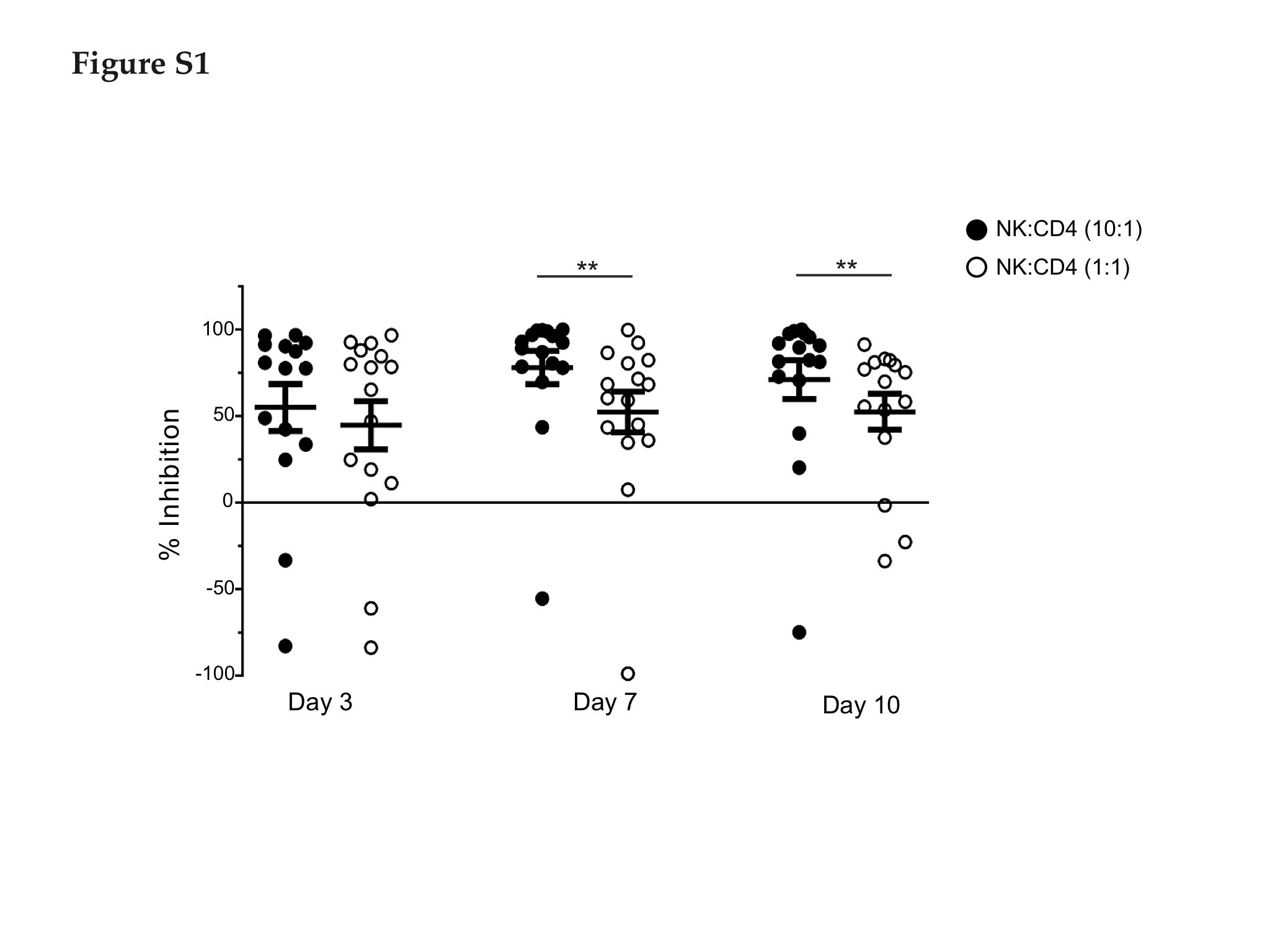

Supplement: Figure S1 — NK cells inhibit HIV replication in autologous HIV infected CD4 (iCD4) cells better at higher NK∶iCD4 cell ratios. The scatter plots show percent inhibition in p24 Gag production in the presence versus the absence of NK cells at NK∶iCD4 cell ratios of 10∶1 and 1∶1 for days 3, 7 and 10 of culture. NK cells from individuals with the following KIR/HLA genotypes were used for this figure: *h/*y+B*57(n = 3), 3DS1+*80I (n = 2), Bw6hmz (n = 4) and other KIR/HLA (n = 8). Each point represents a separate individual. The lines and error bars through the scatter plots show the mean and standard error of the mean for that group. Lines linking groups indicate comparisons where means were significantly different. Friedman tests were used to compare the mean values at different time points for the same NK∶iCD4 cell ratios. Wilcoxon matched pairs tests were used to compare different NK∶iCD4 cell ratios at the same time point. “*” = p<0.05, “**” = p<0.01. (TIFF) [file ppat.1003867.s001.tiff]

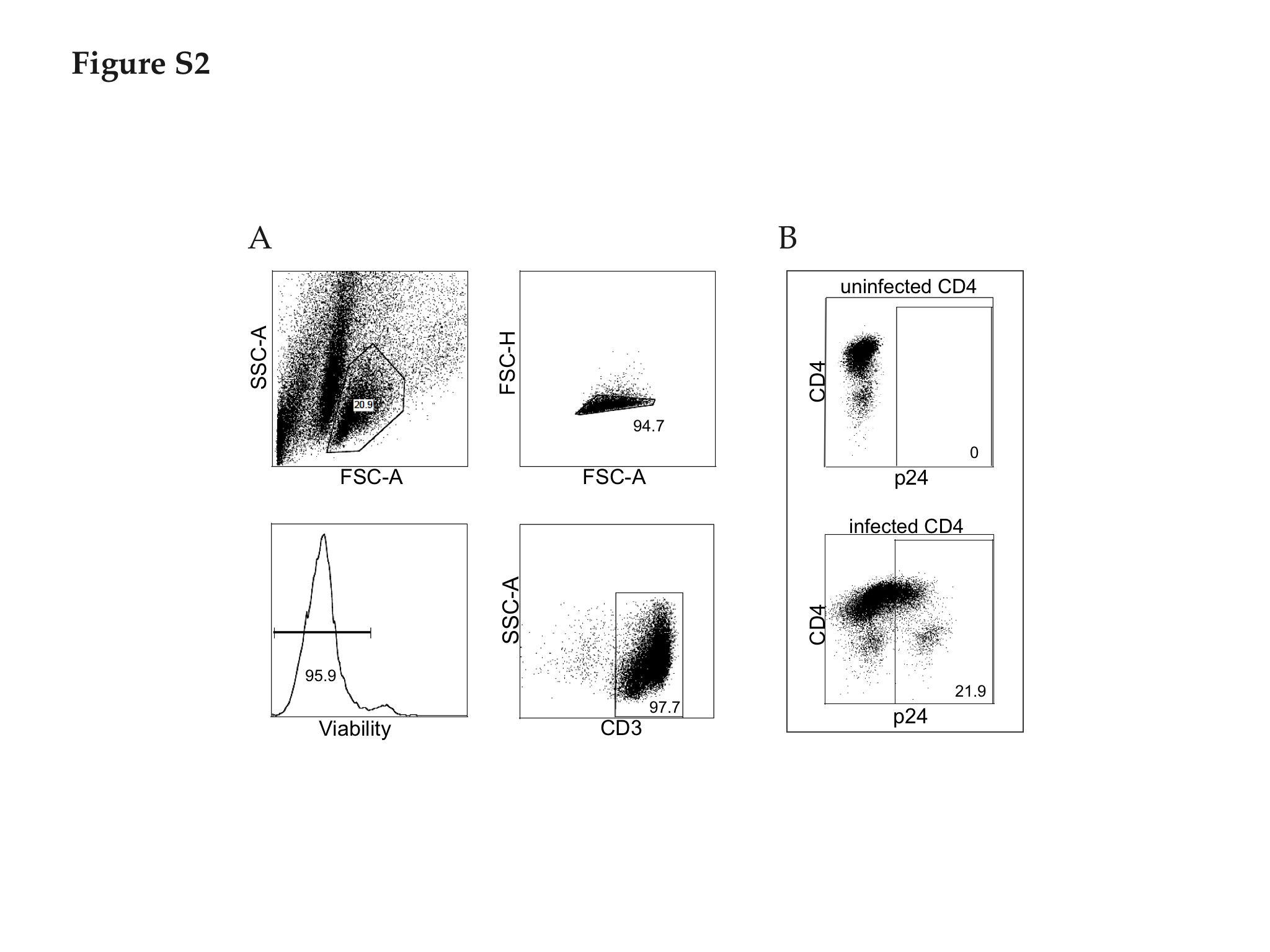

Supplement: Figure S2 — Gating strategy for detection of infected CD4 (iCD4) cells. iCD4 cells co-cultured or not with autologous NK cells were surface stained with anti-CD3 and anti-CD4 antibodies. Cells were then permeabilized and stained for intracellular HIV p24 antigen and Aqua amine reactive dye to distinguish viable and non-viable cells. (A) Live CD3 positive cells were gated on from the lymphocytic singlet population. (B) The percentage of CD3 positive cells that stained for HIV p24 is shown in the boxed area. SSC = side scatter, FSC = forward scatter. (TIFF) [file ppat.1003867.s002.tiff]

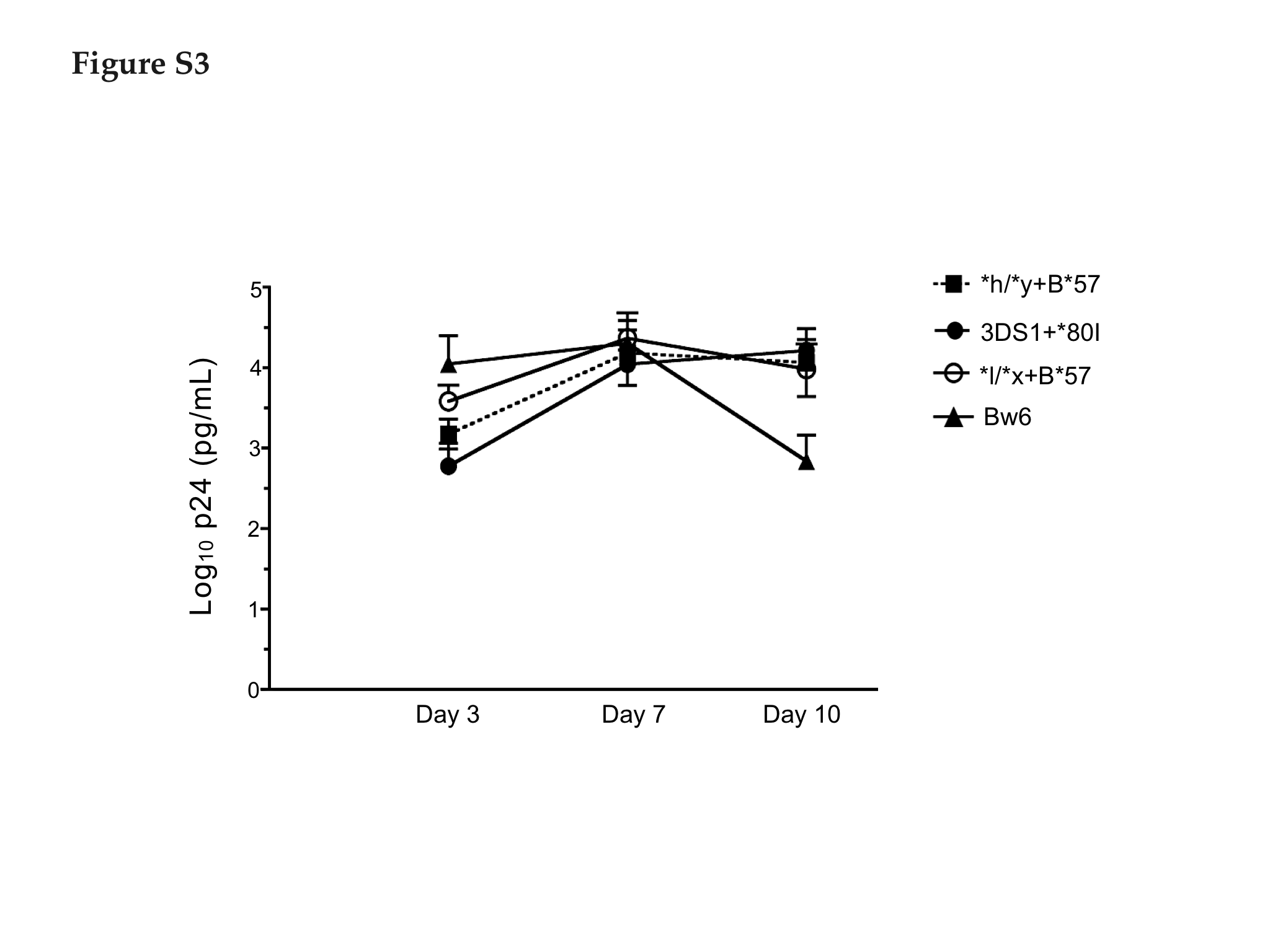

Supplement: Figure S3 — Infected CD4 (iCD4) cells from individuals carrying various KIR/HLA genotypes replicate HIV to similar levels. The line graph depicts the mean change in log10 p24 levels secreted by iCD4 cells. Results were generated using iCD4 from subjects positive for *h/*y+B*57 (n = 7), 3DS1+*80I (n = 9), Bw6hmz (n = 10) and *l/*x+B*57 (n = 4). (TIFF) [file ppat.1003867.s003.tiff]

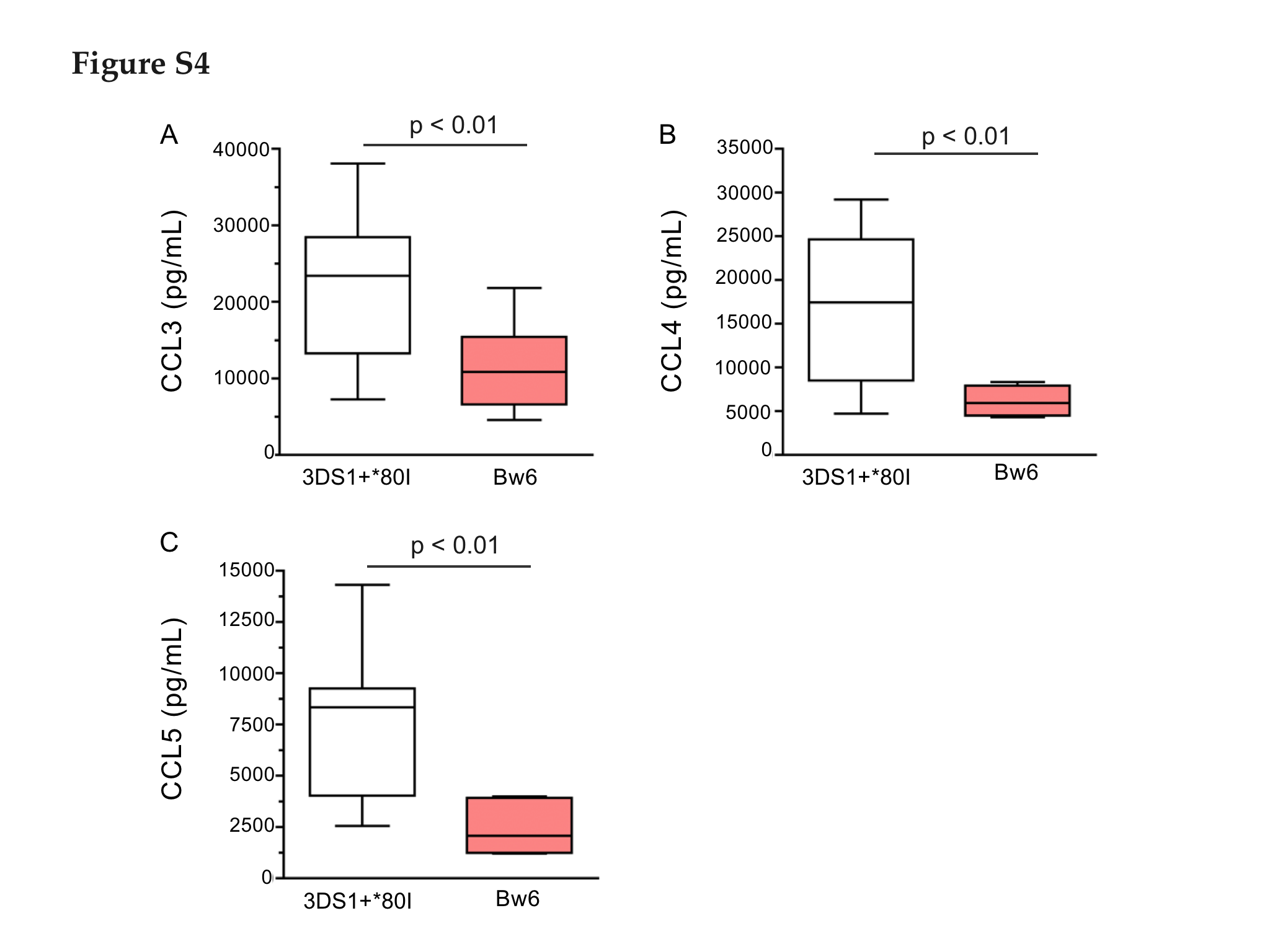

Supplement: Figure S4 — NK cells from 3DS1+*80I carriers secrete more CC-chemokines in response to autologous HIV infected CD4 (iCD4) cells than those from Bw6hmz . Box and whisker plots show levels of CCL3 (A), CCL4 (B) and CCL5 (C) secreted over 3 days into the supernatant of cultures of NK cells and autologous iCD4 cells at a 10∶1 ratio from individuals positive for 3DS1+*80I (n = 12) or from Bw6hmz (n = 10). The line in each box represents the median value, the lower and upper limits of the boxes the 25% and 75% quartiles and the whiskers the minimum and maximum values for each group. P-values are shown over lines linking groups being compared. (TIFF) [file ppat.1003867.s004.tiff]

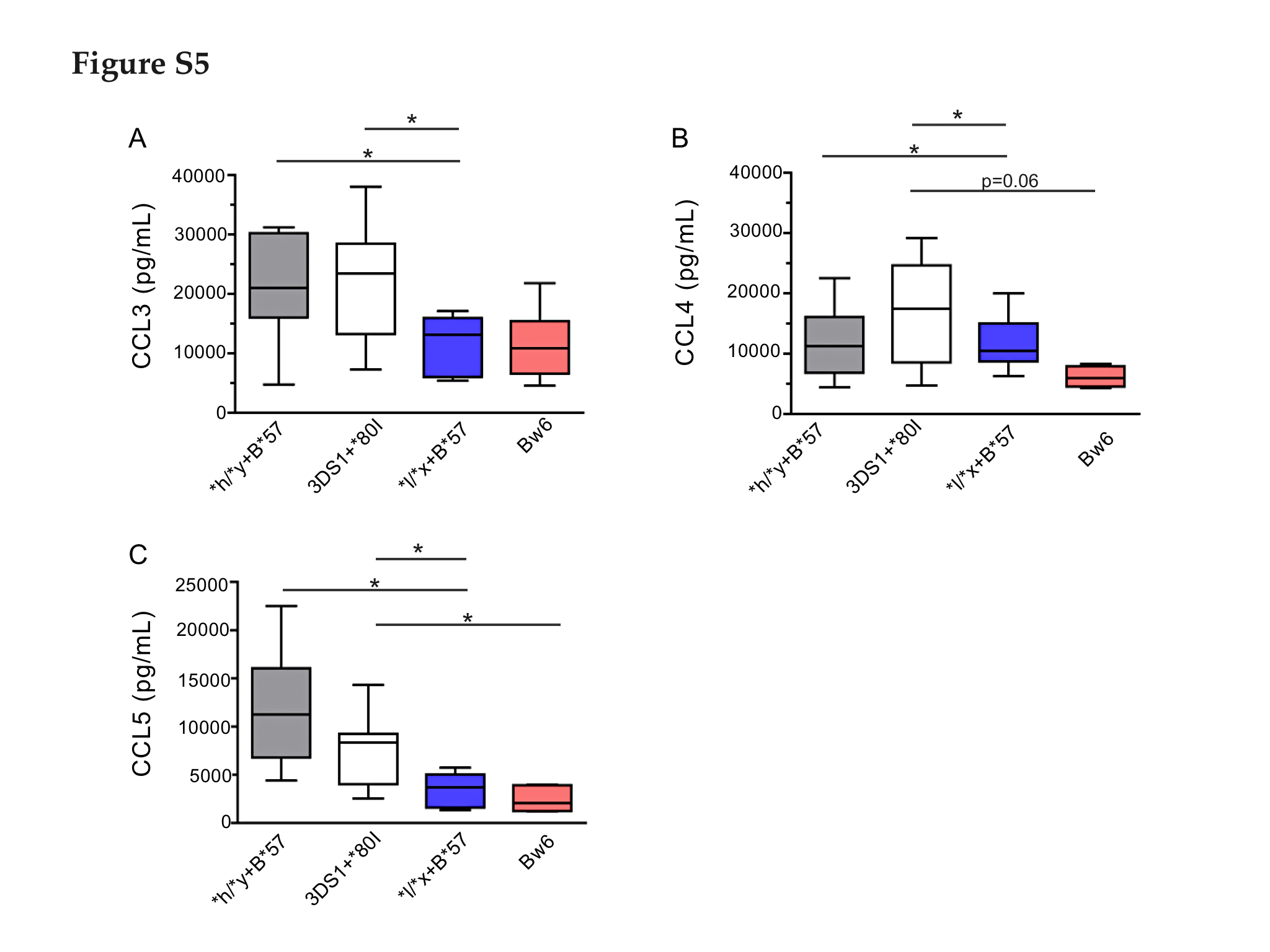

Supplement: Figure S5 — Secretion levels of CC-chemokines from NK cells responding to stimulation with autologous HIV infected CD4 (iCD4) categorized by KIR/HLA genotype. Box and whisker plots show levels of CCL3 (A), CCL4 (B) and CCL5 (C) secreted over 3 days into the supernatant of cultures of NK cells and autologous iCD4 cells at a 10∶1 ratio from individuals positive for *h/*y+B*57 (n = 7) 3DS1+*80I (n = 12) Bw6hmz (n = 10) and *l/*x+B*57 (n = 4). The line in each box represents the median value, the lower and upper limits of the boxes the 25% and 75% quartiles and the whiskers the minimum and maximum values for each group. P-values are shown over lines linking groups being compared. (TIFF) [file ppat.1003867.s005.tiff]

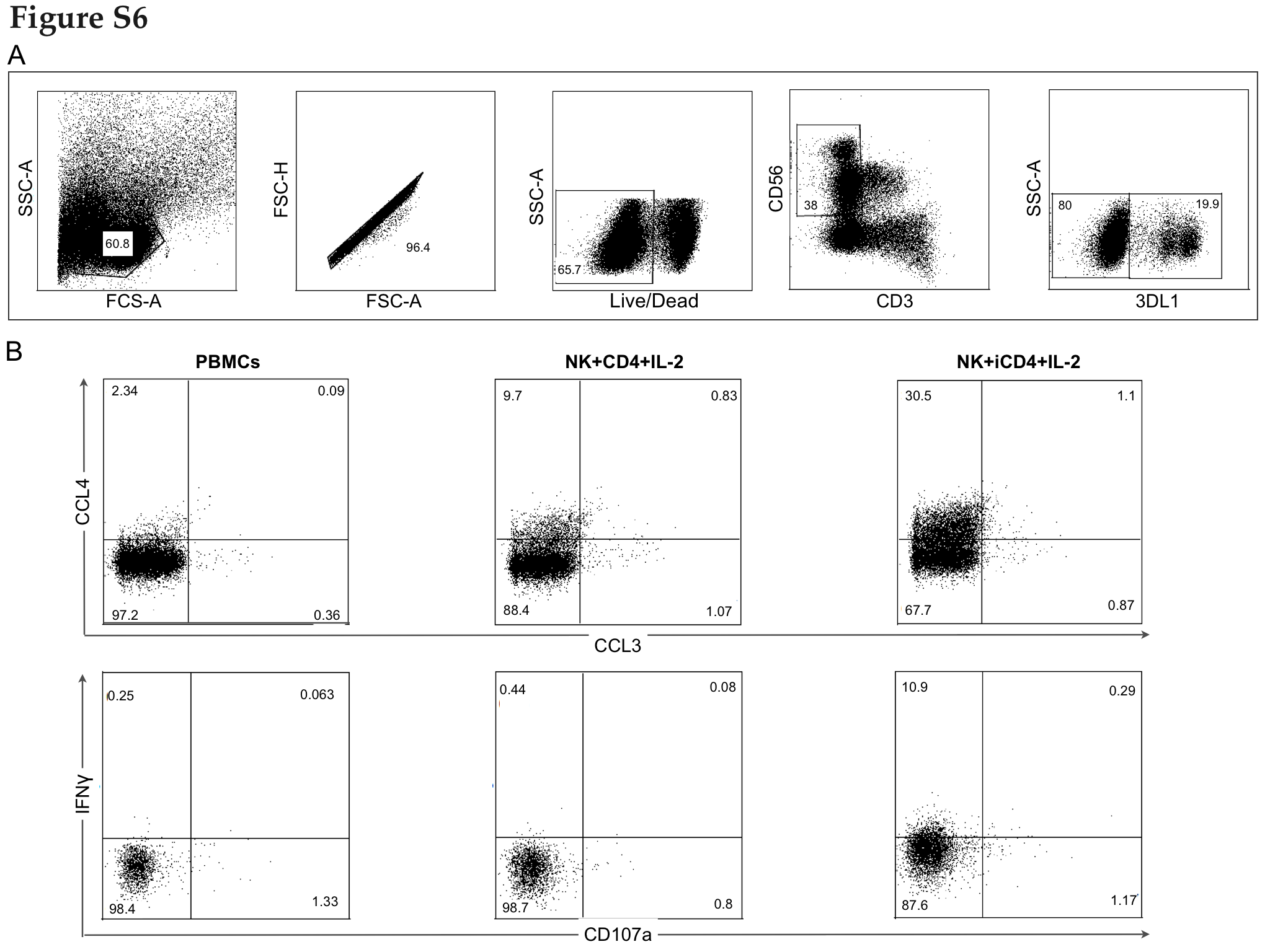

Supplement: Figure S6 — Gating strategy used to assess the percent of functional NK cells stimulated with autologous infected CD4 (iCD4) cells. (A) We used FSC-A and SSC-A to gate on lymphocytes and FSC-A and FSC-H to gate on single cell events from co-cultures of NK cells with autologous CD4 or iCD4 cells. Only live CD3−CD56+ NK cells were included in this analysis. The representative subject shown in this figure carries a KIR3DL1 *l/*x genotype with 1 high and 1 low KIR3DL1 allele. (B) Functional gates were set using unstimulated PBMCs that were gated on the KIR3DL1+ NK cell population using the gating strategy shown in panel A. The percent of CCL3, CCL4, IFN-γ and CD107a positive cells was determined for conditions in which NK and CD4 cells were cultured with IL-2 (negative control) and NK cells and iCD4 cells were cultured with IL-2. NK responses to HIV iCD4 were background subtracted for responses to uninfected CD4 cells. FCS-A = forward scatter area; SSC-A = side scatter area; FSC-H = forward scatter height; PBMC = peripheral blood mononuclear cells. (TIFF) [file ppat.1003867.s006.tiff]

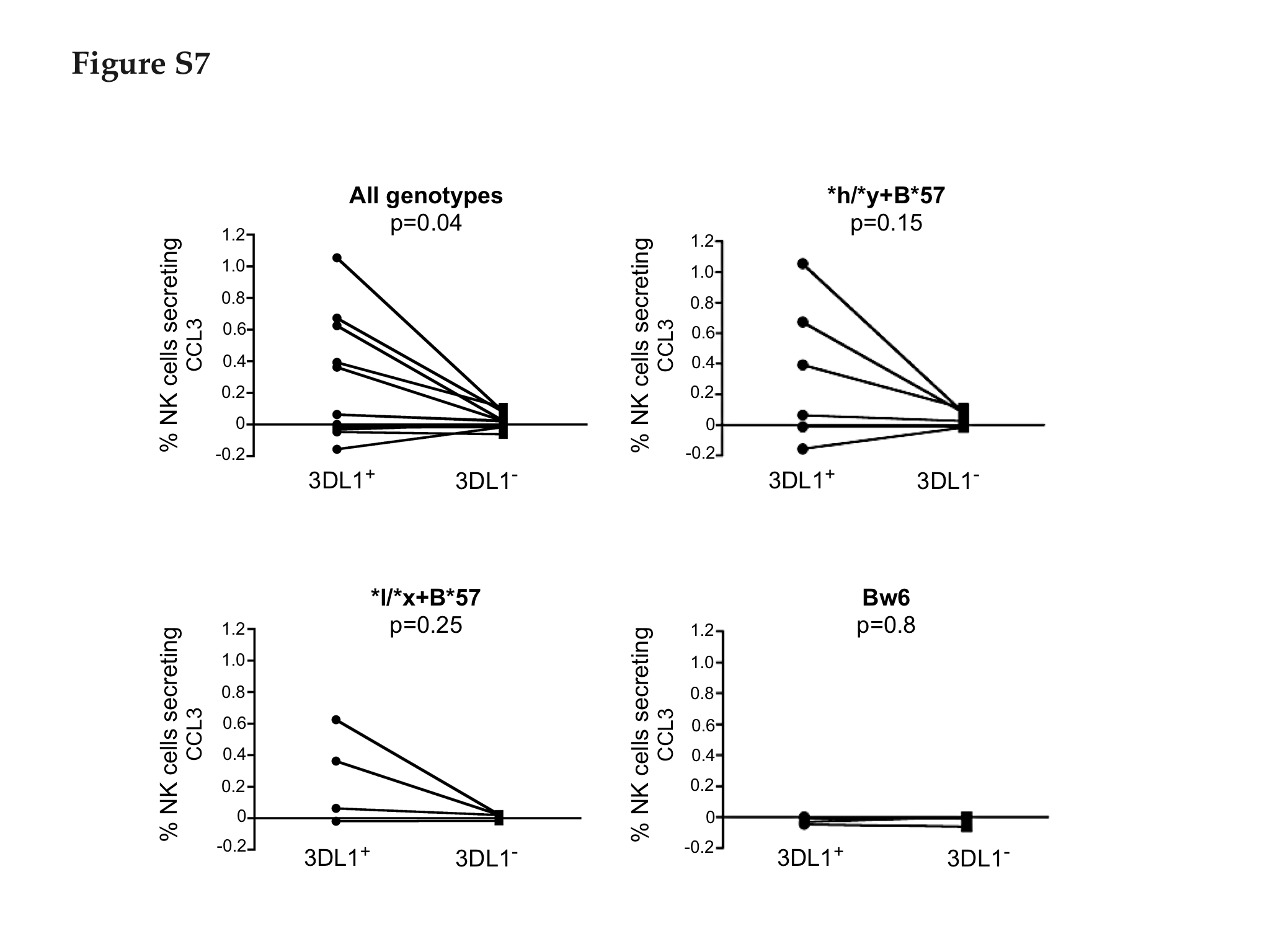

Supplement: Figure S7 — Secretion of CCL3 from KIR3DL1+ (3DL1+) and KIR3DL1− (3DL1−) NK subsets. Paired scatter plots show the percent of 3DL1+ and 3DL1− NK cells secreting CCL3 following stimulation with autologous infected CD4 (iCD4) cells. Shown are results for all individuals tested (upper left panel) and for subjects positive for *h/*y+B*57 (n = 6, upper right) *l/*x+B*57 (n = 4, lower left) and Bw6hmz (n = 4, lower right). The significance of between group differences in the percent of CCL3 secreting cells was tested using a Wilcoxon matched pairs test. P-values for between group comparisons are shown. (TIFF) [file ppat.1003867.s007.tiff]

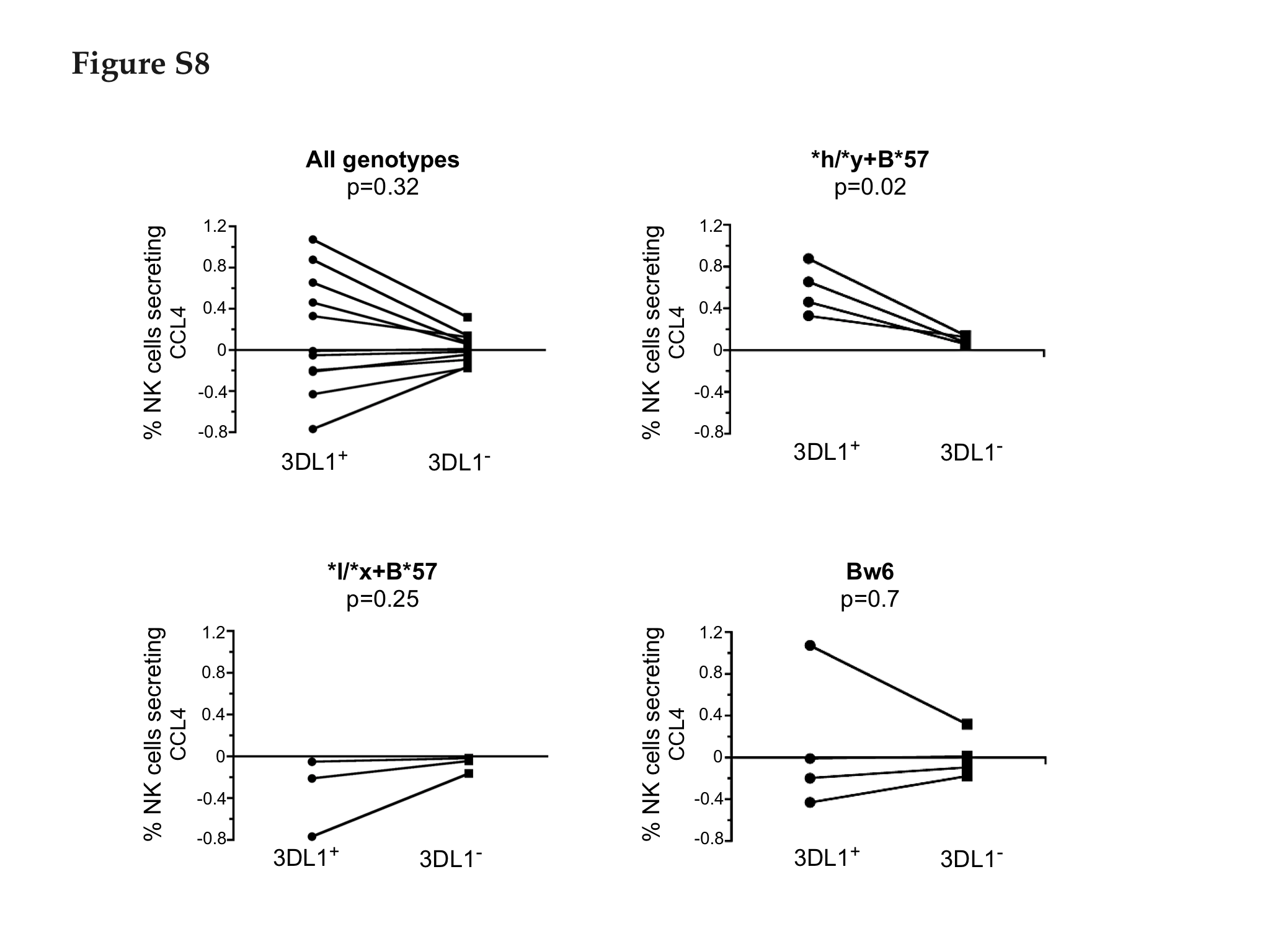

Supplement: Figure S8 — Secretion of CCL4 from KIR3DL1+ (3DL1+) and KIR3DL1− (3DL1−) NK subsets. Paired scatter plots show the percent of 3DL1+ and 3DL1− NK cells secreting CCL4 following stimulation with autologous infected CD4 (iCD4) cells. Shown are results for all individuals tested (upper left panel) and for subjects positive for *h/*y+B*57 (n = 4, upper right) *l/*x+B*57 (n = 3, lower left) and Bw6hmz (n = 4, lower right). The significance of between group differences in the percent of CCL4 secreting cells was tested using a Wilcoxon matched pairs test. P-values for between group comparisons are shown. (TIFF) [file ppat.1003867.s008.tiff]

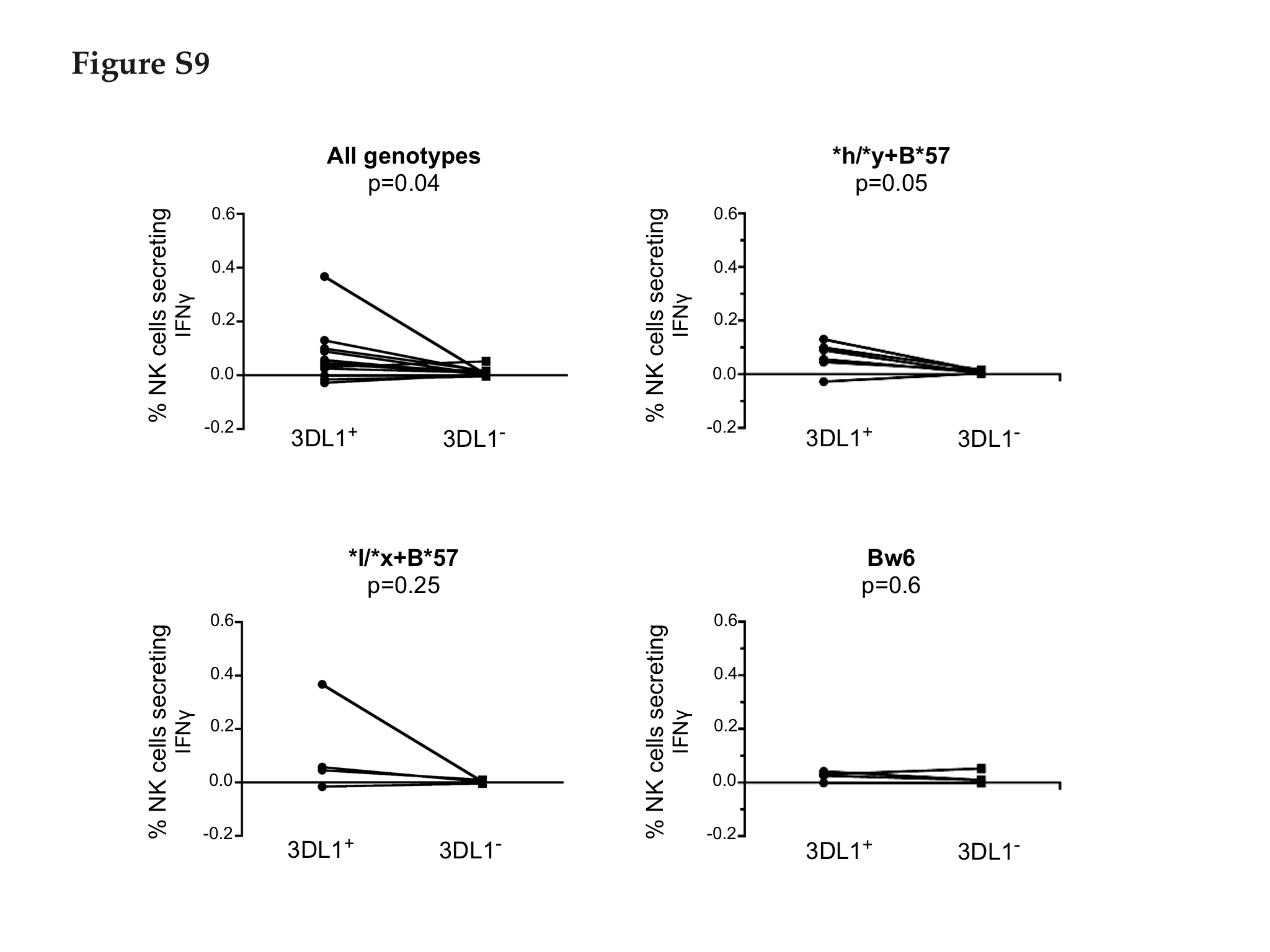

Supplement: Figure S9 — Secretion of IFN-γ from KIR3DL1+ (3DL1+) and KIR3DL1− (3DL1−) NK subsets. Paired scatter plots show the percent of 3DL1+ and 3DL1− NK cells secreting IFN-γ following stimulation with autologous infected CD4 (iCD4) cells. Shown are results for all individuals tested (upper left panel) and for subjects positive for *h/*y+B*57 (n = 6, upper right) *l/*x+B*57 (n = 4, lower left) and Bw6hmz (n = 4, lower right). The significance of between group differences in the percent of IFN-γ secreting cells was tested using a Wilcoxon matched pairs test. P-values for between group comparisons are shown. (TIFF) [file ppat.1003867.s009.tiff]

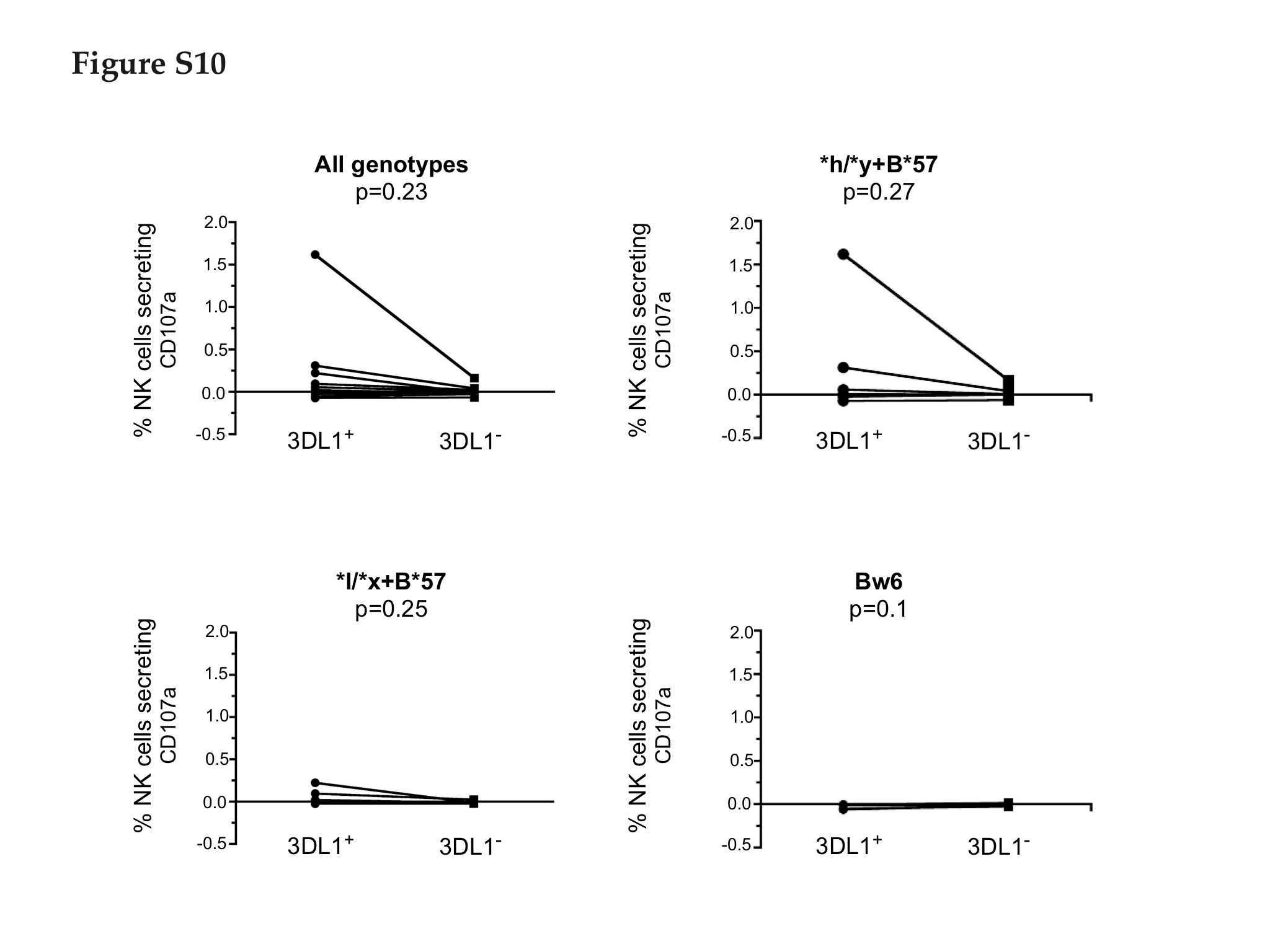

Supplement: Figure S10 — Expression of CD107a in KIR3DL1+ (3DL1+) and KIR3DL1− (3DL1−) NK subsets. Paired scatter plots show the percent of 3DL1+ and 3DL1− NK cells expressing CD107a following stimulation with autologous infected CD4 (iCD4) cells. Shown are results for all individuals tested (upper left panel) and for subjects positive for *h/*y+B*57 (n = 6, upper right) *l/*x+B*57 (n = 4, lower left) and Bw6hmz (n = 4, lower right). The significance of between group differences in the percent of CD107a expressing cells was tested using a Wilcoxon matched pairs test. P-values for between group comparisons are shown. (TIFF) [file ppat.1003867.s010.tiff]
