# Supplementary material for: HIV Protective KIR3DL1/S1-HLA-B Genotypes Influence NK Cell-Mediated Inhibition of HIV Replication in Autologous CD4 Targets
Source: PLoS Pathog. 2014 Jan 16;10(1):e1003867. doi: 10.1371/journal.ppat.1003867 (PMC3894215; doi:10.1371/journal.ppat.1003867)
Supplement: Table S1 — P-values for pair-wise comparisons of CC-chemokine secretion levels by infected CD4 (iCD4) cells stimulated NK cells from individuals categorized by KIR/HLA genotype. The significance of between group comparisons was assessed using Mann-Whitney tests. (DOC) [file ppat.1003867.s011.doc]

Table S1. **P-values for pair-wise comparisons of CC-chemokine secretion levels by infected CD4 (iCD4) cells stimulated NK cells from individuals categorized by *KIR/HLA* genotype**.

| Genotypes compared | CCL3 | CCL4 | CCL5 |
| --- | --- | --- | --- |
| *h/*y+B*57 vs 3DS1+*80I | 0.77 | 0.12 | 0.73 |
| *h/*y+B*57 vs *l/*x+B*57 | 0.38 | 0.92 | 0.15 |
| *h/*y+B*57 vs Bw6hmz | 0.02 | 0.05 | 0.05 |
|  |  |  |  |
| 3DS1+*80I vs *l/*x+B*57 | 0.23 | 0.06 | 0.001 |
| 3DS1+*80I vs Bw6hmz | <0.01 | <0.01 | <0.01 |
| *l/*x+B*57 vs Bw6hmz | 0.61 | 0.03 | 0.15 |
